# Darwin and the biological rhythms

**DOI:** 10.1093/pnasnexus/pgae318

**Published:** 2024-08-27

**Authors:** Tiago G de Andrade, Andrew D Beale

**Affiliations:** Circadian Medicine Center, Faculty of Medicine, Federal University of Alagoas, Campus A.C. Simões, Av. Lourival Melo Mota, s/n - Tabuleiro do Martins, 57072-900, Maceió, Alagoas, Brazil; MRC Laboratory of Molecular Biology, Cambridge Biomedical Campus, Francis Crick Avenue, 01223 267000, Cambridge, United Kingdom

**Keywords:** Darwin, chronobiology, evolution, history of science, circadian

## Abstract

While the formalization of chronobiology as a scientific discipline occurred in the mid-20th century, the exploration of rhythmic phenomena has a longer history, notably exemplified by De Mairan's observations of *Mimosa pudica* in darkness in 1729. In this historical narrative, Charles Darwin is known for his investigations into the “sleep movements” of plants. Nevertheless, the complete scope of Darwin's exploration of biological rhythms remains incompletely understood. Through a detailed examination of Darwin's writings, meticulous observations, experiments, and conceptualizations, we unveil a depth of engagement that surpasses his widely acknowledged work on plants, revealing a more extensive interest in and insight into biological rhythms than traditionally recognized.

**Significance statement**

This manuscript unveils a previously overlooked facet of Charles Darwin's scientific endeavors, revealing his extensive engagement with biological rhythms beyond his well-known work on plant movements. Through meticulous review of Darwin's writings, including letters, notebooks, and other publications, the research brings to light a more profound and expansive interest in biological rhythms than previously recognized. Notably, certain records in Darwin's notes appear to foreshadow concepts and experimental observations later explored by chronobiology in the succeeding century.

## Introduction

Charles Darwin is commonly referenced in the historical reviews of chronobiology for his later work on the “sleep movements” in plants, published in 1880 alongside his son Francis. In *The Power of Movements in Plants*, Darwin (1) meticulously investigated the rhythmic opening and closing of leaves in different species (Supplementary Information Appendix 1.1). While considerable attention has been given to Darwin's exploration of plant movements, including during the opening address of the landmark 1960 Cold Spring Harbor Symposium on Biological Clocks (2), a more extensive appreciation of the breadth and depth of his thoughts and contributions to the field of biological rhythms is lacking.

In fact, Darwin's interest in plant movements and daily timing traces back to at least 1838, evident in his references to Augustin de Candolle's work on sensitive plants (3) (Supplementary Information Appendix 1.2). Examining his notebooks from the same period reveals early contemplations on whether sensitive plants might naturally close at specific times daily, alongside explorations of memory in plants and associations with the closing of leaves (4, 5) (Supplementary Information Appendix 1.3–4). These entries underscore Darwin's early curiosity about the nature and origin of periodic movements in plants, suggesting a more profound interest in biological rhythms.

In a letter to L. Jennys in 1847, Darwin expressed amusement at having a list detailing the order of appearance of plants and animals around him, emphasizing the need for precise temporal resolution. This highlights Darwin's fascination with the periodic phenomena of animals and plants, indicating a desire for improved temporal precision in his observations (6) (Supplementary Information Appendix 1.5).

This work aims to illuminate and contextualize Darwin's thoughts on biological rhythms, shedding light on a potentially overlooked aspect of his interests in these phenomena. Revisiting Darwin's work with a chronobiological perspective, through his books, letters, and notebooks (7), reveals that his exploration of biological rhythms extended beyond plants to also include the animal kingdom. It also suggests that he likely attributed a higher importance to this feature than previously acknowledged, expanding his curiosity to encompass the broader realm of biological timekeeping, including inheritance and adaptive value. Instead of strictly adhering to a chronological structure, this paper presents Darwin's approach to biological rhythms as thematic explorations with broader implications for chronobiology. It spans from a primarily observational endeavor to a deeper, more speculative, and experimental exploration of “periodical phenomena.” This includes illustrating Darwin's wide-ranging interest in periodic events, with an emphasis on his recognition of time as a crucial variable in observing natural phenomena; highlighting Darwin's acknowledgment of the variability of rhythmic traits within populations; exploring Darwin's contemplation of the adaptive value of rhythmicity in plants and other organisms; delving into how Darwin interpreted the potential impact of rhythmic phenomena on speciation; discussing Darwin's recognition of the hereditary nature of biological rhythms in various organisms, along with his speculation of potentially associated endogenous mechanisms; and showcasing his controlled experiments with worms, which bear resemblance to modern chronobiological approaches. Quotes related to these topics are highlighted in the main text, with further context and fuller quotes found in the accompanying Supplementary Information Appendix.

### Time as a bias

Darwin, during his extensive voyages on the *HMS Beagle*, demonstrated a keen awareness of periodic phenomena in both geology and biology across various time scales. His observations spanned diurnal and nocturnal habits in animals, seasonal migrations, cyclical changes associated with reproduction, hibernation patterns, and tidal rhythms, among other temporal aspects (Supplementary Information Appendix 2.1–2.123). Crucially, Darwin exhibited a genuine preoccupation with time as a bias in interpreting biological phenomena (Supplementary Information Appendix 2.124–2.132). Darwin's meticulous approach encompasses his acknowledgment of the potential seasonality effect on sampling. In the context of Cirripedia, he examined numerous specimens from diverse localities and at different times of the year in order to observe traces of ova or ovaries (8) (Supplementary Information Appendix 2.124–2.125).

This emphasis on the impact of time is further evidenced by instances such as when Darwin decided to rigorously examine *Orchis morio* (green-winged orchid) (Supplementary Information Appendix 2.126). Over 23 consecutive days, he scrutinized the flowers at various times, considering factors like hot sunshine, rain, and different hours of the day (9). Additionally, in his investigation of whether a secretion from a carnivorous plant could affect enamel and dentine, Darwin maintained a disciplined routine, examining each succeeding day at the same hour (10) (Supplementary Information Appendix 2.127). In his correspondences, he explicitly emphasized the importance of meticulous observation to ensure accurate documentation of events, such as the activities of nocturnal or diurnal insects visiting plants at specific times (11) (Supplementary Information Appendix 2.128–2.131).

This conscientiousness is not merely a “side effect,” but rather is a testament to Darwin's meticulous methodology, as highlighted by Ayala (12). In a letter to *The Gardeners’ Chronicle* in 1855, Darwin sought specific details about a peculiar “shell rain” in the Isle of Wight, inquiring about the exact hour and day of the phenomenon (13) (Supplementary Information Appendix 2.132). This meticulous attention to temporal details may suggest that Darwin's approach extended beyond mere observation, potentially influencing a deeper interpretation of biological rhythms.

### Biological rhythms are variable in populations

Genetic variation, the driving force for natural selection, extends its influence to biological rhythms. Today, we understand that polymorphisms in clock genes, clock-controlled genes, and those genes involved in synchronization mechanisms contribute to the variability in biological rhythms within populations [reviewed in (14)]. Beyond genetics, developmental and environmental factors, as well as sex and age, further contribute to the interindividual variation in circadian physiology and behavior (14–16).

Remarkably, Darwin's recognition of variability extended to biological rhythms, as observed in plants and animals under domestication. The modifications in the habitual “periods” of different families within the same species, including the time of reproduction, the acquisition of capacity, and the hour of roosting, reflect a parallel with the diverse and variable habits observed in plants. This suggests for him a correlation between “corporeal” and plant-based periodical habits, further emphasizing the extent of variability in biological rhythms (17, 18) (Supplementary Information Appendix 3.1–3.4).

Darwin may have acknowledged individual-level variability in diurnal preferences within himself (Supplementary Information Appendix 3.5). There is evidence to suggest that he leaned toward being a “morning type,” consistently rising early and regarding the morning hours as his most productive. This inclination is supported by Francis Darwin, who observed Charles Darwin's early-rising habits and his tendency to initiate scientific visits in London by 10 in the morning (6) (Supplementary Information Appendix 3.6–3.7).

It is clear therefore that Darwin was appreciative of time as a biological variable and the variance of rhythmic traits. Did he recognize the adaptive value of biological rhythms as well?

### The adaptive value of biological rhythms

Darwin's thoughts on the selective value of biological rhythms were downplayed in the pivotal 1960 Cold Spring Harbor Symposium on Biological Clocks (2). During the symposium's opening address, Bunning claimed that “it never occurred to Darwin that the endogenous diurnal rhythm might have a selective value” (2). However, recent perspectives challenge this notion (19).

Darwin explicitly proposed that “the movements depend on innate causes, and are of an adaptive nature” (1). He suggested that the nocturnal position of leaves may be an adaptation against radiative heat loss, moisture loss and to exclude nocturnal insects ill-adapted for fertilization (1) (Supplementary Information Appendix 4.1–4.7). Some of these hypotheses have been experimentally validated, such as the contribution of leaf movements to heat loss (20).

However, Darwin's recognition of the adaptive nature of biological rhythms was not limited to plants (Supplementary Information Appendix 4.8–4.25). Darwin's view encompassed the broader impact of periodical phenomena, including those of daily, seasonal, and yearly time scales, on the adaptation and evolution of diverse organisms. In a draft for the “Instinct” chapter of *On the Origin of Species* written in 1857 and preserved in the final 1859 version, Darwin argued that changes in “instinct” within the same species “at different periods of life or time of the year” could be preserved by natural selection (21) (Supplementary Information Appendix 4.10). Indeed, in many passages, Darwin recognized the periodic nature of strategies to improve reproduction. This included proposing that the time of pairing in animals coincides with a period of full vigor, emphasizing the importance of favorable conditions for reproduction (21) (Supplementary Information Appendix 4.12), as well as a suggestion that some flowers emit odor chiefly in the evening to be fertilized by crepuscular or nocturnal insects (10) (Supplementary Information Appendix 4.9).

Darwin noted that species with limited access to food or inadequate migration capabilities face challenges in population growth during periods of food scarcity. For him, migration becomes a crucial strategy for birds to cope with periodic food shortages, and the absence of migratory capabilities hinders certain species from attaining large populations during seasons of food scarcity (22) (Supplementary Information Appendix 4.15). In *The Descent of Man, and Selection in Relation to Sex*, Darwin proposed that the habit of molting twice a year may have evolved through natural selection, allowing birds to cast off inconvenient ornaments during the winter (23) (Supplementary Information Appendix 4.8). These observations are coupled with the notion that De Candolle's war of nature is fundamentally periodic (22) (Supplementary Information Appendix 4.26–4.40).

Finally, in one of the most insightful passages in *The Descent of Man*, Darwin speculates that some periodic phenomena or traits with fixed durations in vertebrates may be reminiscent of ancestral biological rhythms from marine organisms adapted to tidal cycles (Supplementary Information Appendix 4.41–4.43):

Now it is a mysterious fact that in the higher and now terrestrial Vertebrata, as well as in other classes, many normal and abnormal processes have **one or more whole weeks as their periods** (*gestation of mammals, the duration of fevers, the hatching of eggs*); this would be rendered intelligible if the Vertebrata are **descended from an animal allied to the existing tidal Ascidians.** (Author emphasis) (23) (Supplementary Information Appendix 4.41)

Lunar or semi-lunar periods have indeed been proved to be important for humans and other animals (24, 25), which may align with Darwin's suggestion.

Since Darwin, the adaptive value of circadian rhythms has been demonstrated in numerous elegant experiments [reviewed in (26)]. Some of the strongest evidence comes from circadian resonance experiments across various organisms: cyanobacteria, plants, flies, hamsters, and mice in semi-natural conditions. These studies have shown that resonant and nonresonant endogenous circadian periods can alter the competitiveness of the organism (27–32).

### Temporal isolation as a mechanism for speciation

If time is of selective value, it may impact speciation. Darwin's original notion of speciation occurring within populations sharing the same geographical area faced significant challenges from modern evolutionary biologists, notably Ernst Mayr in the 20th century (33). Mayr contested Darwin's concept of sympatric speciation, which involved “temporal niches” or isolation in the timing of reproduction, as a source of variation and a mechanism for evolutionary divergence. Darwin argued that even among slow-breeding animals, intercrosses within the same area did not necessarily hinder natural selection, as distinct varieties could persist due to factors like occupying different habitats, breeding at slightly different seasons, or displaying preferences for specific mating partners (34) (Supplementary Information Appendix 5.1–5.7). He observed that in plants, a difference in the flowering period played a crucial role in maintaining the distinctness of varieties, exemplified by various types of maize and wheat (18) (Supplementary Information Appendix 5.8–5.11).

In recent years, Darwin's observations and propositions have been supported. The concept of allochrony, a type of sympatric speciation driven by “temporal niches,” has garnered support from mathematical models, experimental studies, and empirical evidence across various time scales and families (35–40). Notably, variations in clock genes have been identified in several cases, providing molecular insights into the temporal aspects of speciation and adaptive radiation (36, 41). This reaffirms Darwin's pioneering ideas and underscores the importance of temporal isolation over the day scale as well as over the annual scale as a mechanism contributing to the diversity of life.

### Inheritance and endogenous nature of periodical phenomena

The advancement of chronobiology crucially hinged on the acknowledgment that biological rhythms are endogenous (and by extension, inherited) and not merely a byproduct of external cyclical factors. While De Candolle emphasized the intrinsic nature of leaf movements in plants as early as the 19th century, widespread acceptance of endogeneity only occurred in the early 20th century through the groundbreaking experiments of Erwin Bunning, Anthonia Kleinhoonte, and Wilhelm Pfeffer (19, 42). In his writings, Darwin considered both the inheritance and endogenous nature of rhythmicity, positions that extended from his work on other behavioral and physiological phenomena.

Darwin's perspective on the inheritance of periodicity is revealed in early writings. In a draft for *On the Origin of Species* written in 1858, which was later included in the final version published the following year, Darwin explored periodicity, probably regarding migratory instincts and reproductive behavior in birds:

Changes of instinct may sometimes be facilitated by the same species having different instincts at different periods of life, or at different seasons of the year, or when placed under different circumstances. (34) (Supplementary Information Appendix 6.2)

In 1842, within the initial draft of his species theory, Darwin's early writings also suggested a broad view of the extent of periodicity in animals as well as plants, stating that “Habits purely corporeal, **breeding season, time of going to rest, vary and are hereditary**, like the analogous habits of plants which vary and are inherited” (Author emphasis) (17) (Supplementary Information Appendix 6.2). Darwin's fascination with the circannual “instinct of migration” in birds and mammals, as observed in the “transandantes” sheep in Spain, underscores his recognition of the inherited nature of this behavior as early as 1844 (17) (Supplementary Information Appendix 6.3). These early expressions precede the publication of *The Power of Movement in Plants* by approximately 4 decades.

We can conjecture that De Candolle's experiments influenced this opinion, as Darwin was aware of them as early as 1838 (Supplementary Information Appendix 1.2). However, for Darwin to extend such conclusions to various phenomena and animal species, there must have been additional influences at play. This could, in part, be ascribed to the insights of plant and animal breeders, a theme consistent with other propositions in evolutionary theories. As an example, he drew on Bechstein's observations regarding the hereditary nature of singing habits in nightingales (43) (Supplementary Information Appendix 6.4).

In *The Descent of Man*, Darwin devoted a section to the “inheritance at corresponding seasons of the year” (Supplementary Information Appendix 6.5). According to Darwin,

Although I do not know that this tendency to change the color of the coat during different seasons is transmitted, yet it probably is so, as all shades of color are strongly inherited by the horse. Nor is this form of inheritance, as limited by the seasons, more remarkable than its limitation by age or sex. (23) (Supplementary Information Appendix 6.5)

He went as far as suggesting that the migratory instinct is so potent that it can ultimately override the maternal instinct (23) (Supplementary Information Appendix 6.6). Darwin could have considered seasonal (and other) rhythms as a natural aspect of human behavior as well. Another passage from this work reinforces this view:

Man is subject like other mammals, birds, and even insects, to that **mysterious law**, which causes certain normal processes, such as gestation, as well as the maturation and duration of various diseases, to **follow lunar periods.** (Author emphasis) (23) (Supplementary Information Appendix 6.7)

However, recognizing the inheritance of biological rhythms does not necessarily imply assuming the existence of an endogenous, autonomous, clock. What Darwin appears to suggest is an association between external periodic phenomena and some form of inherited instinct. In other words, rhythms could emerge as a result of the influence of external, periodic elements, in a predisposed organism, akin to an “hourglass” mechanism previously discussed (44). It remains unclear if Darwin approached conceptualizing an endogenous oscillator as we now understand true biological rhythms. Despite his demonstrated curiosity about what might provoke these rhythms, especially migratory instincts, he explicitly expressed frustration regarding his ignorance of the underlying mechanism:

Difficult though it may be to conceive how animals either intelligently or instinctively come to know a given period. (45) (Supplementary Information Appendix 6.8)

It is notable that Pfeffer, a contemporary of Darwin, initially contested endogeneity, attributing sleep movements to the aftereffects of light and darkness. Darwin's writings suggest that he sided with De Candolle when he explicitly challenged Pfeffer's views on inheritance and endogeneity:

Pfeffer denies such inheritance; he attributes the periodicity when prolonged for a day or two in darkness, to “Nachwirkung,” or the after-effects of light and darkness. But we are unable to follow his train of reasoning. (1) (Supplementary Information Appendix 6.9)

The aforementioned observation on lunar periods in *The Descent of Man* leads to a citation from Thomas Laycock, a British physician who published a series of papers in 1842 about “the general law of vital periodicity,” applied to animals and humans, including medical conditions (46–49). Laycock formulated a classification for the cyclical process into: (i) inherited in the system (“esoteric”), (ii) promoted by the environmental cycles (“exoteric”), and (iii) mixed (“endexoteric”). According to Laycock, “esoteric” processes “go on to a great extent independently of external periodic influences” (47). Drawing from his understanding of both De Candolle's and Laycock's works, Darwin may therefore have assumed the inherited and endogenous nature of biological rhythms across various organisms. Following Laycock's assumptions on “esoteric” rhythms, he may have conceived some preliminary concept of a self-sustained biological clock. While we cannot be certain of this, it is intriguing to speculate from his writings if he considered the biological process behind rhythmic phenomena.

Darwin acknowledged that animals could assess the intervals between recurrent events, raising inquiries about the mechanisms involved (23) (Supplementary Information Appendix 6.10). His ponderings might find clarity in the distinction he made between “instinct” and “faculty” (Supplementary Information Appendix 6.11–6.13). Concerning migration, Darwin proposed separating the instinct to proceed in a particular direction from the means by which animals discern directions and initiate travel at specific periods.

[…] with migratory birds, it is a wonderful instinct which urges them at certain times of the year to direct their course in certain directions, but it is a faculty by which they know the time and find their way. (17) (Supplementary Information Appendix 6.11)

In another foundational essay for his evolutionary theories, written in 1844, he marveled at animals' ability to accurately judge time, citing instances like a hawk visiting monasteries every 3 weeks, showcasing an awareness of a recurring period (17) (Supplementary Information Appendix 6.11). In contemplating migration, Darwin ventured into the realm of instinctive desires associated with corporeal sensations, suggesting a link between the tendency to move and an innate understanding of time (17) (Supplementary Information Appendix 6.11–6.12). Francis Darwin, in interpreting his father's ideas, emphasized the challenge of distinguishing between faculties and instincts, underscoring the intricate connection between actions and underlying capabilities (Supplementary Information Appendix 6.13). For instance, the “bird of passage has the faculty of finding its way and the instinct to put it in action at certain periods” (17). Darwin also subtly alluded to the existence of a sensory mechanism influencing the organism in response to external factors. According to Darwin, true instincts, characterized by invariability and nonimprovement during an individual's mature age, were associated with specific states of the body or particular times of the year or day (17) (Supplementary Information Appendix 6.12).

Therefore, while Darwin did not explicitly recognize the endogenous nature of periodicity and expressed his incomprehension of its mechanism (45) (Supplementary Information Appendix 6.8), throughout his writings he made numerous allusions to, and suggestions of, a timing system that is both inherited and shows recurrence.

### A pioneer for chronobiological experiments?

In his final work, along with his son Francis, Darwin conducted observational and intervention experiments in earthworms, revealing some intriguing chronobiological facets (50) (Supplementary Information Appendix 7). Darwin's firsthand and correspondence-based knowledge provided detailed insights into the temporal behavior of worms (Supplementary Information Appendix 7.1–7.5):

Worms are nocturnal in their habits, and at night may be seen crawling about in large numbers, but usually with their tails still inserted in their burrows […] During the day they remain in their burrows, except at the pairing season, when those which inhabit adjoining burrows expose the greater part of their bodies for an hour or two in the early morning. (50)

To better investigate their habits without disruption, Darwin and his son took precautions: “This difficulty led my son Francis and myself to observe worms in confinement during several nights by the aid of a dim light, while they dragged the leaves of the above-named pines into their burrows” (50) (Supplementary Information Appendix 7.6).

Darwin's interest in how worms perceive and react to light, despite being “destitute of eyes,” led to experiments with different light intensities, durations, and colors. He concluded that “From the foregoing facts, it is evident that light affects worms by its intensity and duration” (50) (Supplementary Information Appendix 7.7). Moreover, Darwin experimented with the effect of light at different times of day and described what we now could recognize as a potential “phase shift” in activity onset: “If in the evening the pots were illuminated before the worms had come out of their burrows, they failed to appear” (50) (Supplementary Information Appendix 7.8). Although he simply described the event as an example of reactive sensitivity to light, Darwin notably made a distinction between a simple reflex act and some inner form of predisposition, recognizing that light exerts different effects at different times due to variations in the animal's nervous system:

But the **different effect which a light produced on different occasions**, and especially the fact that a worm when in any way employed and in the intervals of such employment, whatever set of muscles and ganglia may then have been brought into play, is **often regardless of light**, are opposed to the view of the sudden withdrawal being a simple reflex action. (Author emphasis) (50) (Supplementary Information Appendix 7.9)

Similar observations were made regarding the worm's reaction to vibration, with Darwin noting that “their sensitiveness to jars varied much at different times,” although he did not explicitly mention the time of day (Supplementary Information Appendix 7.10). Darwin's experiments with light hinted at investigations into synchronization mechanisms conducted in the 20th century and ongoing today. A particularly unequivocal description of a circadian rhythm emerged when the worms were kept in a simulated constant, “free-running” condition, controlling light and potentially temperature (north-east window) variations:

Their withdrawal into their burrows during the day appears, however, to have become an habitual action; **for worms kept in pots covered by glass-plates, over which sheets of black paper were spread, and placed before a north-east window, remained during the day-time in their burrows and came out every night**; and they continued thus to act for a week. (Author emphasis) (50) (Supplementary Information Appendix 7.11)

This observation, akin to De Mairan's work (51) for *Mimosa*, foreshadowed later research in the field of chronobiology (19). Interpreting “habitual action” as an inherited and adapted form of (periodical) behavior, evolved to “escape extreme danger from the many diurnal animals which prey on them” (50) (Supplementary Information Appendix 7.11), aligns with Darwin's broader evolutionary insights and the endogenous nature of circadian rhythms.

It was not until the latter half of the 20th century that the endogenous foundations of the circadian clock were unveiled, with molecular mechanisms discovered only in the late 20th century [(52, 53); reviewed in (54)]. Progress on the biological mechanisms of annual migrations and other seasonal behaviors is recent and ongoing (55–57). Biological rhythms must synchronize to zeitgebers in order for the animal to be properly adapted to its ecological environment (58, 59). This synchronization relies on the relationship between “sensors,” like the retina, and central pacemakers such as the suprachiasmatic nucleus and the pineal gland (60, 61), as well as the timing mechanisms in myriad peripheral tissues and cells (62). The significant discoveries of these biological pacemakers began to emerge in the late 1960s, nearly a century after Darwin's initial contemplations (19).

## Conclusion

This study reveals a more profound engagement of Darwin with biological rhythms than previously acknowledged. The historical contexts that might have prompted Darwin to consider biological rhythms and his interpretations on this matter still necessitate exploration. Furthermore, a detailed analysis of the potential influence of these observations of biological rhythms on the development of Darwin's evolutionary theories warrants further investigation. The groundwork laid by Darwin's predecessors, particularly Laycock, also merits further exploration in historical reviews on basic and applied chronobiology. In fact, Laycock anticipated many modern concepts in the field (46). Although Darwin's grandfather, Erasmus Darwin, delved into “periodical diseases” in the 18th century, highlighting the recognition of rhythms in diseases, Charles Darwin did not explicitly reference this work in his writings.

The evidence presented in this manuscript indicates that Darwin, throughout his life as a gentleman naturalist, maintained a persistent interest in cyclical events. His meticulous observations and experimental endeavors demonstrate this interest, expanding the scope of his contributions beyond the study of sleep movements in plants.

## Supplementary Material

pgae318_Supplementary_Data

## Data Availability

All data used for this review can be accessed at Darwin Online (https://darwin-online.org.uk/).
