# Supplementary material for: Darwin and the biological rhythms
Source: PNAS Nexus. 2024 Aug 27;3(8):pgae318. doi: 10.1093/pnasnexus/pgae318 (PMC11348560; doi:10.1093/pnasnexus/pgae318)
Supplement: pgae318_Supplementary_Data [file pgae318_supplementary_data.zip › Andrade&Beale_Darwin_Supporting Information(SI)_Revised-1.pdf]

Supporting Information (SI) Appendix
Darwin and the biological rhythms
Tiago G de Andrade, Andrew D Beale

1. Introduction

1. Anyone who had never observed continuously a sleeping plant, would naturally suppose that the leaves moved only in the evening when going to sleep, and in the morning when awaking; but he would be quite mistaken, for we have found no exception to the rule that leaves which sleep continue to move during the whole twenty-four hours; they move, however, more quickly when going to sleep and when awaking than at other times. http://darwin-online.org.uk/content/frameset?keywords=to%20in%20evening%20the%20leaves%20moved%20going%20when%20only%20sleep&pageseq=420&itemID=F1325&viewtype=text
2. Decandolle has a chapter on sensitive plants. https://darwin-online.org.uk/content/frameset?itemID=CUL-DAR124.-&keywords=has%20sensitive%20plants%20a%20chapter%20decandolle%20on&viewtype=text&pageseq=165
3. Would a sensitive plant if irritated at one time every day naturally close at that time after a long period. http://darwin-online.org.uk/content/frameset?keywords=one%20irritated%20plant%20at%20time%20a%20sensitive%20if%20would%20every&pageseq=148&itemID=CUL-DAR125.-&viewtype=text
4. The memory of Plants, must be association, — a certain round of actions take place every day, & closing of the leaves, comes on from want of stimulus, after certain other actions, & hence becomes associated with them. — The establishment of this principle of Association will help my theory of sensitive Plants. http://darwin-online.org.uk/content/frameset?keywords=principle%20of%20establishment%20he%20this%20association&pageseq=29&itemID=CUL-DAR126.-&viewtype=text
5. I think it is very amusing to have a list before one's eyes of the order of appearance of the plants and animals around one; it gives a fresh interest to each fine day. There is one point I should like to see a little improved, viz. the correction for the clock at shorter intervals. Most people, I suspect, who like myself have dials, will wish to be more precise than with a margin of three minutes. http://darwin-online.org.uk/content/frameset?keywords=list%20amusing%20very%20is%20to%20i%20before%20a%20have%20think%20it&pageseq=373&itemID=F1452.1&viewtype=text

2. Time as a bias

Rhythms in Geology

1. The tides in most cases reach the cliffs only for a short time twice a day, and the waves eat into them only when they are charged with sand or pebbles; for there is reason to believe that pure water can effect little or nothing in wearing away rock. . http://darwin-online.org.uk/content/frameset?keywords=most%20reach%20time%20in%20for%20a%20twice%20tides%20the%20cases%20cliffs%20day%20only%20short&pageseq=381&itemID=F387&viewtype=text
2. I shd think all the above was accounted for by uniformity & non uniformity of conditions. Periods of drought & damp alone makes astonishing difference. — clear sky makes heat in day & also at night effect animal kingdom also. http://darwin-online.org.uk/content/frameset?pageseq=1&itemID=CUL-DAR73.100-102&viewtype=text

3. Dry season return periodically after every. http://darwin-online.org.uk/content/frameset?pageseq=1&itemID=CUL-DAR38.835a&viewtype=text
4. With a clear sky it froze sharply every night. http://darwin-online.org.uk/content/frameset?pageseq=1&itemID=F1925&viewtype=text
5. the hour of day makes a considerable difference in the difficulty of crossing. http://darwin-online.org.uk/content/frameset?pageseq=1&itemID=F1925&viewtype=text
6. In the months, included between the end of March beginning of April & beginning of October, it proceeds towards the NE: & from November October to end of March again it returns to the SW. — Carb of Lime [crusty] 2.7 This movement is probably owing to a similar periodical change in the prevailing swell of the sea; the direction of the wind remaining nearly constant. Lieut Evans also informs me, that during the six years he has resided on the Isld. he has observed that, in the months of October & November of each month year, a hard calcareous incrustation appears, on the lava rocks of the beach, near the settlement. The incrustation is hard, white, resembling the nature of the thick shells of molluscous animals & is composed of successive thin layers. It appears gradually & disappears in the same manner, within the time specified [ie] it varies now only coats the tidal rock from close to the settlement & in no other part of this Isld; — it varies in quantity in the different years. —. http://darwin-online.org.uk/content/frameset?pageseq=1&itemID=CUL-DAR38.936-953&viewtype=text
7. Found great numbers of a species of Limnœa3 adhering to aquatic plants in a lake situated between Mandetiba & Lagoa Araruama: The water was then fresh.— but the inhabitants affirmed that periodically once an year it became salt & sometimes oftener.— The period most probably in which the SW winds prevail; Is not this fact curious, that fresh water shells should survive an inundation of salt water? In the neighbouring Lagoon, Balani were adhering to the rocks. http://darwin-online.org.uk/content/frameset?pageseq=1&itemID=F1840&viewtype=text
8. Monday 23rd Walked with Maccormick to Flag Staff Hill. — We passed over an extended plain of table land. — There was scarcely one green leaf on the whole tract, yet large flocks of goats, together with some cattle, contrive to live. — It rains but very seldom in this country & when it does a mass of vegetation springs up; this soon drys up & withers: & upon such miserable sort of hay the animals exist: at present it has not rained for a year, & I suppose will not till the proper time next year, viz. November & October. — At these periods the island is very unhealthy: one ship some years past lost six of its junior officers. — A little to the North of the hill, we found a very curious ravine, not much above 30 yards across. — about 200 feet high. We with some difficulty found one single path at the very end, where we descended. — In this wild dell we found the building places of many birds. — Hawks & Ravens & the beautiful Tropic bird were soaring about us: a large wild cat bounded across & reached its den before Maccormick could shoot it. — The place seemed formed for wild animals: large blocks of rocks, entwined with succulent creepers & the ground strewed over with bleached bones of Goats would have been a fine habitation for a Tiger. http://darwin-online.org.uk/content/frameset?pageseq=1&itemID=F1925&viewtype=text
9. In summer these torrents are of course quite impassable, the scene of violence which their beds show at this time of year may give one some idea of their strength & fury. http://darwin-online.org.uk/content/frameset?pageseq=1&itemID=F1925&viewtype=text
10. (a) In the form of many of the small Islands in the Bay, I noticed a fact, which I believe is of common recurrence, but is not by me very easily explicable. — At low water the figure of the island is a hill [sketch] surrounded by a low level ledge of naked rock, which is only covered at nearly the time of high water. http://darwin-online.org.uk/content/frameset?pageseq=1&itemID=CUL-DAR37.802-811&viewtype=text
11. within a hundred yards of the beach at spring tides. the fresh water rises within 18 «or 12» inches of the general level: the water ebbs & flows in these wells. about 4 ft. in which

thickness. the whole is Calcareous sand. partly «slightly» consolidated agglutinated. http://darwin-online.org.uk/content/frameset?keywords=the%20at%20spring%20beach%20of%20yards%20hundred%20a%20tides%20within&pageseq=1&itemID=CUL-DAR41.40-57&viewtype=text

12. The central dead parts of the masses of «of the Millepora» net works «& the towers of masses of Astrea» are coated & raised by a Corallina. — The channels are coated with the same substance & have a little sand. at the bottom. to seaward. similar lumps. for a distance of 10-20 yards might be seen beneath the water. beyond this for another 20 yards there was green water «notable» water & then came the Blue. — Where we stood. the masses of Astrea were from 4 — 8 ft in diameter, but irregular in figure. & the channels about 6 ft deep: The Astrea, was on its surface to a depth of 3 or 4 inches was dead. further inland a greater depth was dead. to seaward. without doubt the whole surface. was living. – It is a beautiful instance how short a time is sufficient to kill them: at this time under such favourable circumstances the dead parts were awash. The Astrea. extends laterally & must soon fill up the channels. — Specimens. ([blank space]) will show. a layer. additional on the sides as compared to the top. — The Millepora. http://darwin-online.org.uk/content/frameset?pageseq=1&itemID=CUL-DAR41.40-57&viewtype=text
13. This substance evidently bears exposure. for some time to the air «Yet. which is very curious. must require a breaking sea:» — instantly the surface of the Astrea dies. it is occupied by Corallina — http://darwin-online.org.uk/content/frameset?keywords=surface%20the%20of%20astrea%20dies%20instantly&pageseq=9&itemID=CUL-DAR41.40-57&viewtype=text
14. The highest part of the reef, is on the outer edge where the sea breaks; at the outer edge; on the windward side of the Island at low water a few small points may [word deleted] be seen uncovered I am told, that at low water on the windward side a large part reef at such time is quite dry, & generally in every part of few small points patches of Coral rock may be then seen uncovered. Between this outer line & the shore beach the water is so shallow, so that people, avoiding the deep holes & channels, can commonly wade out at low water to fish. — generally at low water in most parts the tide falls at low water. http://darwin-online.org.uk/content/frameset?pageseq=1&itemID=CUL-DAR38.882-901&viewtype=text
15. There are some arguments which strike the mind with force. — the exact yearly rise of the great rivers prove better than any meterological table the precise periods over immense areas. (& the counterbalancing variations) of rain. = The Bulk of sediment [daily] yearly brought down by every torrent proves the decay atmospheric of the most solid rocks.—The grand cliffs of a thousand feet in height, of the solid lavas.—proportionally high to age. (we do not wonder to see tertiary plains consumed) Where slope [plainly] indicates former boundary. http://darwin-online.org.uk/content/frameset?pageseq=1&itemID=F1583e&viewtype=text
16. in rivers that have periodical overflowings"2 & in the Orinoca. the layer is said to occur in the parts periodically washed by the waters. — It In my case the it is easy to believe that the tidal action corresponds to the periodical overflowings. & the action of the surf. to that of a rapid or a cataract. — (a> In all the cases the rocks are granitic, but as such formations contain such distinct minerals as quartz. felspar. mica & Hornblende. I cannot believe their composition can have produce any effect. — Probably all that is required is a solid surface not subject to rapid decomposition. There is another coincidence in the intertropical climate, which likewise is common to the calcareous varnish at Ascension (? at Serpentine at St Pauls). but it Berzelius, at the request of Humboldt. http://darwin-online.org.uk/content/frameset?pageseq=1&itemID=CUL-DAR32.9-14&viewtype=text
17. On the evening of the succeeding day we reached the Rio de las Vacas, which is considered the worst stream in the Cordillera to cross. As all these rivers have a rapid and short course,

and are due to the snow melted by the sun's heat, the hour of the day makes a considerable difference in their volume. In the evening the stream is muddy and full, but about daybreak it becomes both clearer and much less impetuous. This we found to be the case with the Rio Vacas, and in the morning we crossed it with little difficulty.. http://darwin-online.org.uk/content/frameset?pageseq=1&itemID=F10.3&viewtype=text

18. The main line of beach is directed N.E. and S.W.; Lieutenant Evans informs me, that during the six months included between the 1st of April and the 1st of October, the sand accumulates towards the N.E. extremity, and during the other six it travels back again towards the S.W. end. This periodical movement is due to a change in the direction of the swell, which is influenced by the general direction of the trade-wind, during the two periods of the year. Lieutenant Evans also informs me that during the six years he has resided on this island, he has always observed, that in the months of October and November, when the sand commences travelling towards the S.W., the rocks which are situated at that end of the long beach, become coated by a white, thick, and very hard calcareous layer. I saw portions of this remarkable deposit, which had been protected by an accumulation of sand. In the year 1831 it was much thicker than during any other period. It would appear that the water, charged with calcareous matter by the disturbance of a vast mass of calcareous particles, only partially cemented together, deposits this substance on the first rocks against which it impinges. But the most singular circumstance is, that in the course of a couple of months this layer is either abraded or redissolved, so that after that period, it entirely disappears. It is curious thus to trace the origin of a periodical incrustation on certain isolated rocks, to the motion of the earth with relation to the sun; for this determines the atmospheric currents, which give the direction to the swell of the ocean, and this acts on the arrangement of the sea-beach, and this again on the quantity of calcareous matter held in solution by the waters of the neighbouring sea. http://darwin-online.org.uk/content/frameset?pageseq=1&itemID=F10.3&viewtype=text
19. These droughts to a certain degree seem to be periodical; I was told the dates of several others, and the intervals were about fifteen years. A tendency to periodical droughts is, I believe, common in most dry climates :† such certainly is the case in Australia. Captain Sturt says they return after every ten and twelve years, and are then followed by excessive rains, which gradually become less and less, till another drought is the consequence. http://darwin-online.org.uk/content/frameset?pageseq=1&itemID=F10.3&viewtype=text
20. They occur only within the limits of tidal action; and as the rivulet slowly trickles down, the surf must supply the polishing power of the cataracts in the great rivers. In the same manner, the rise and fall of the tide probably answers to the periodical inundations. http://darwin-online.org.uk/content/frameset?pageseq=1&itemID=F10.3&viewtype=text
21. Gauchos assured me, that in the middle of the dry summer, this stream, at the same time with the Colorado, has periodical floods; which can only originate in the snow melting on the Andes. It is extremely improbable that a stream, so small as the Sauce then was, should traverse the entire width of the continent; and indeed, if it were the residue of a large river, its waters, as in other ascertained cases, would be saline. During the winter we must look to the springs round the Sierra Ventana as the source of its pure and limpid stream. I suspect the plains of Patagonia, like those of Australia, are traversed by many water-courses, which only perform their proper parts at certain periods. Probably this is the case with the water which flows into the head of Port Desire, and likewise with the Rio Chupat, on the banks of which masses of highly cellular scoriæ were found by the officers employed in the survey. As it was early in the afternoon when we arrived, we took fresh horses, and a soldier for a guide, and started for the Sierra de la Ventana. http://darwin-online.org.uk/content/frameset?pageseq=1&itemID=F10.3&viewtype=text
22. The bed of pebbles in the valley west of the town, is intersected by a second valley joining it as a tributary, but even this valley appears much too wide and flat-bottomed to have been formed by the small quantity of water, which falls only during one short wet season; for at

other times of the year, these valleys are absolutely dry. http://darwin-online.org.uk/content/frameset?pageseq=1&itemID=F272&viewtype=text

23. In the wet season, the whole, or a part, of the salt is dissolved, being redeposited during the succeeding dry season. At this period, the appearance of the snow-white expanse of salt crystallized in great cubes, is very striking. http://darwin-online.org.uk/content/frameset?pageseq=1&itemID=F273&viewtype=text
24. These fragments, from being repeatedly caught in the ice and stranded with violence, and from being every summer exposed to common littoral action, will generally be much worn; and from being driven over rocky shoals, probably often scored. From the ice not being thick, they will, if not drifted out to sea, be landed in shallow places, and from the packing of the ice be sometimes driven high up the beach, or even left perched on ledges of rock. http://darwin-online.org.uk/content/frameset?pageseq=1&itemID=F1677&viewtype=text

Rhythms in Biology

25. in the evening some species of frogs make a concert no ways unpleasant. this as the night advances is accompanied by the endless cry of the Cicadas. http://darwin-online.org.uk/content/frameset?pageseq=1&itemID=F1840&viewtype=text
26. Zoonomia, p. 437: "Master A. about nine years old, had been seized at seven every morning for ten days with uncommon fits . . . he began to complain of pain about his navel, or more to the left side, and in a few minutes had exertions of his arms and legs like swimming. He then for half an hour hunted a pack of hounds; as appeared by his hallooing. . . ."http://darwin-online.org.uk/content/frameset?pageseq=1&itemID=CUL-DAR125.-&viewtype=text
27. It is a very tame, most quiet, solitary little bird, and like the English robin (Sylvia rubecula) it is usually most active early in the morning and late in the evening. http://darwin-online.org.uk/content/frameset?pageseq=1&itemID=F8.11&viewtype=text
28. At M: Video, every animal was hybernating (Vide P 120) when the mean Temp: was 58°.4 & in the day Therm: often rising to 70°.— The difference of Latitude between the latter & this place is four degrees or 240 miles; Thus showing how much the general annual Temp: affects the degree at which animals reassume their living process.— http://darwin-online.org.uk/content/frameset?pageseq=1&itemID=F1840&viewtype=text
29. Bell remarks that, at the approach of sleep, or of a fainting-fit, or of death, the pupils are drawn upwards and inwards; and he believes that "when we are wrapt in devotional feelings, and outward impressions are unheeded, the eyes are raised by an action neither taught nor acquired;" and that this is due to the same cause as in the above cases.26 That the eyes are upturned during sleep is, as I hear from Professor Donders, certain. With babies, whilst sucking their mother's breast, this movement of the eyeballs often gives to them an absurd appearance of ecstatic delight; and here it may be clearly perceived that a struggle is going on against the position naturally assumed during sleep. But Sir C http://darwin-online.org.uk/content/frameset?pageseq=1&itemID=F1142&viewtype=text
30. During the night colours are not visible, and there can be no doubt that the nocturnal moths, taken as a body, are much less gaily decorated than butterflies, all of which are diurnal in their habits. http://darwin-online.org.uk/content/frameset?pageseq=1&itemID=F944&viewtype=text
31. The Tucutuco (Ctenomys Brasiliensis) is a curious small animal, which may be briefly described as a Gnawer, with the habits of a mole. It is extremely numerous in some parts of the country, but is difficult to be procured, and never, I believe, comes out of the ground. It throws up at the mouth of its burrows hillocks of earth like those of the mole, but smaller. Considerable tracts of country are so completely undermined by these animals that horses, in passing over, sink above their fetlocks. The tucutucos appear, to a certain degree, to be gregarious: the man who procured the specimens for me had caught six together, and he said this was a common occurrence. They are nocturnal in their habits; and their principal food is the roots of plants, which are the object of their extensive and superficial burrows. This animal

is universally known by a very peculiar noise which it makes when beneath the ground http://darwin-online.org.uk/content/frameset?keywords=is%20the%20brasiliensis%20curious%20animal%20ctenomys%20small%20tucutuco%20a&pageseq=73&itemID=F20&viewtype=text

32. Where this animal is abundant, it may be heard at all times of the day, and sometimes directly beneath one's feet. When kept in a room, the tucutucos move both slowly and clumsily, which appears owing to the outward action of their hind legs; and they are quite incapable, from the socket of the thigh-bone not having a certain ligament, of jumping even the smallest vertical height. They are very stupid in making any attempt to escape; when angry or frightened they uttered the tucu-tuco. Of those I kept alive, several, even the first day, became quite tame, not attempting to bite or to run away; others were a little wilder. http://darwin-online.org.uk/content/frameset?keywords=both%20in%20a%20move%20kept%20the%20slowly%20tucutucos%20when%20room&pageseq=64&itemID=F14&viewtype=text
33. The ground at the place where we stopped for the night was incrusted with a layer of sulphate of soda, and hence, of course, was without water. Yet many of the smaller rodents managed to exist even here, and the tucutuco was making its odd little grunt beneath my head, during half the night. http://darwin-online.org.uk/content/frameset?pageseq=1&itemID=F59&viewtype=text
34. The remarkable length of the nectary, containing much nectar, the white colour of the conspicuous flower, and the strong sweet odour emitted at night, all show that this plant depends for it fertilisation on the larger nocturnal Lepidoptera. http://darwin-online.org.uk/content/frameset?pageseq=1&itemID=F800&viewtype=text
35. The fact of a larger proportion of white flowers smelling sweetly may depend in part on those which are fertilised by moths requiring the double aid of conspicuousness in the dusk and of odour. So great is the economy of nature, that most flowers which are fertilised by crepuscular or nocturnal insects emit their odour chiefly or exclusively in the evening. Some flowers, however, which are highly odoriferous depend solely on this quality for their fertilisation, such as the night-flowering stock (Hesperis) and some species of Daphne; and these present the rare case of flowers which are fertilised by insects being obscurely coloured. http://darwin-online.org.uk/content/frameset?pageseq=1&itemID=F1249&viewtype=text
36. Man is subject like other mammals, birds, and even insects, to that mysterious law, which causes certain normal processes, such as gestation, as well as the maturation and duration of various diseases, to follow lunar periods. http://darwin-online.org.uk/content/frameset?pageseq=1&itemID=F937.1&viewtype=text
37. They feed by day, and do not wander far from their burrows. http://darwin-online.org.uk/content/frameset?pageseq=1&itemID=F20&viewtype=text
38. On the other hand, cats, from their nocturnal rambling habits, cannot be matched, and, although so much valued by women and children, we hardly ever see a distinct breed kept up; such breeds as we do sometimes see are almost always imported from some other country, often from islands. http://darwin-online.org.uk/content/frameset?pageseq=1&itemID=F380&viewtype=text
39. deaths owing to time of day. http://darwin-online.org.uk/content/frameset?pageseq=1&itemID=CUL-DAR35.354-356&viewtype=side
40. I have never seen a bee or any other diurnal insect. http://darwin-online.org.uk/content/frameset?pageseq=1&itemID=F1706&viewtype=text
41. The flowers are very small and inconspicuous, but emit a strong musky smell, especially at night. http://darwin-online.org.uk/content/frameset?pageseq=1&itemID=F800&viewtype=text
42. I have suspected that the testes are periodically renewed, or at least redeveloped from an undistinguishable condition. http://darwin-online.org.uk/content/frameset?pageseq=1&itemID=F339.2&viewtype=text

43. Many species, belonging to many genera have very wide ranges, compared with most marine animals;1 & this is important for us in allowing extensive migration during the cool period. Prof. Dana states that the sub-torrid shores of Natal, Japan, & even the Sandwich islands have several identical species & several representative species not found in the intervening torrid seas; & Prof. Dana doubts, though granting the possibility of wide migration, whether these species could possibly have passed from the southern to the northern zones. http://darwin-online.org.uk/content/frameset?keywords=compared%20most%20to%20genera%20have%20ranges%20very%20wide%20many%20species%20belonging%20with&pageseq=571&itemID=F1583&viewtype=text
44. From this fact, I infer that whatever nocturnal insect (for I never saw an insect visit the plants by day) haunts this orchis had ceased its visits, as indeed might be inferred from the extreme terminal flowers of the three plants which had never been covered, retaining their pollen-masses. http://darwin-online.org.uk/content/frameset?pageseq=1&itemID=F1583&viewtype=text
45. other birds (& this seems common kind migration of America) migrate singly flying few miles every day & generally by night—one bird which is strictly diurnal, migrates singly by night.—others in flocks, kind of migration quite different in species of same genus, these birds seem clearly directed by kind of country. http://darwin-online.org.uk/content/frameset?pageseq=1&itemID=F1583&viewtype=text
46. p 3832. case of summer Duck. Dendromessa sponsa Sw. with a few acorns in both speci shot at different times. http://darwin-online.org.uk/content/frameset?pageseq=1&itemID=CUL-DAR73.144-146&viewtype=text
47. This curious and unique structure answers, I believe, the same purpose as the four convex, hardish, crenated buttons on the posterior thoracic cirri in Alcippe, which are likewise unique in that genus. I observed that in some specimens the teeth had been worn quite blunt, but the teeth and hairs are periodically moulted and renewed, together with the whole œsophagus. http://darwin-online.org.uk/content/frameset?pageseq=1&itemID=F339.2&viewtype=text
48. Consequently the points generally show signs of severe attrition, but they are periodically and often replaced, at each exuviation, by new and much sharper points. The external membrane over the whole animal, excepting the horny disc which covers the rostral face of the peduncle, is very thin and transparent; it is periodically and often moulted, as may be inferred from the many old lines of junction round the edges of the horny disc […] At each exuviation, the external membrane with the dentated hardened orifice, the lateral bars, the inner tunic of the sack with its bars, are all moulted, together with the usual integuments of the animal's body. New and sharp triturating points are thus periodically formed for the work of excavation. As the animal grows, the old sclerodermic plates, all joined together, are moulted, and new ones, also all joined together, of a larger size, are formed beneath. Now let us imagine the growth to be more gradual but yet periodical, and the new and larger sclerodermic plates, when formed under the old ones, to adhere firmly to them; the older plates would thus be prevented from becoming confluent, and instead of being all moulted together, as is now the case, they would be almost continually separated from each other, owing to the almost continuous increase in size of the new underlying plates. This species, is very easily killed by brackish water, as are some other species, whilst B. improvisus and eburneus can flourish in it; and at the Falkland Islands, I saw Elminius Kingii attached to rocks at the mouth of a fresh-water brook, so as to be covered by pure water during the ebb of each tide. http://darwin-online.org.uk/content/frameset?keywords=new%20increase%20continuous%20the%20size%20to%20underlying%20of%20in%20almost%20owing&pageseq=148&itemID=F339.2&viewtype=text
49. Growth of the whole Shell, and its Microscopical Structure.—The opercular valves are added to along their basal margins alone;* the animal's body, together with the several muscles, becoming attached at each period of growth lower and lower down to the valves; this no

doubt is effected by the absorption of the upper surfaces of the muscles, and the formation of new fasciæ on their lower surfaces. The opercular membrane, which, though thin and flexible, forms part of the general outer surface of the animal as much as does any portion of the rigid shell, with which indeed it is strictly homologous, is periodically moulted, together with the integuments of the whole included animal. The new opercular membrane is of course each time formed a little larger than the old one. http://darwin-online.org.uk/content/frameset?pageseq=1&itemID=F339.2&viewtype=text

50. Concluding Remarks.—The males from the absence of a mouth (and no doubt of a stomach), must necessarily be short-lived, and, I suppose, are periodically replaced by fresh males.* In one instance, the remnants of the two * It is possible, though opposed to all analogy, that the females may be short-lived, and breed only once, in which case the males would not have to be periodically replaced. http://darwin-online.org.uk/content/frameset?pageseq=1&itemID=F339.1&viewtype=text
51. * It deserves notice, that in the class Crustacea, both in the Lerneidæ and in the Cirripedia, the males more closely resemble the larvæ, than do the females; whereas amongst insects, as in the case of the glow-worm in Coleoptera, and of certain nocturnal Lepidoptera, it is the female which retains an embryonic character, being worm-like or caterpillar-like, without wings. But in all these cases, the male is more locomotive than the female. http://darwin-online.org.uk/content/frameset?pageseq=1&itemID=F339.1&viewtype=text
52. This species is diurnal in its habits, and may be daily seen under a scorching sun, crawling over the parched and loose sand. http://darwin-online.org.uk/content/frameset?pageseq=1&itemID=F8.19&viewtype=text
53. I have considered all the Fuegian specimens as belonging to one species. It is possible, however, that the larger specimens may prove distinct, though the differences, which are not apparently important, more probably arise from the period of the year at which they were gathered, as noticed above in Mr. Darwin's notes. http://darwin-online.org.uk/content/frameset?pageseq=1&itemID=F1671&viewtype=text
54. he little owl (Athene cunicularia), which has been so often mentioned, on the plains of Buenos Ayres exclusively inhabits the holes of the bizcacha; but in Banda Oriental it is its own workman. During the open day, but more especially in the evening, these birds may be seen in every direction standing frequently by pairs on the hillock near their burrows. If disturbed they either enter the hole, or, uttering a shrill harsh cry, move with a remarkably undulatory flight to a short distance, and then turning round, steadily gaze at their pursuer. Occasionally in the evening they may be heard hooting. I found in the stomachs of two which I opened the remains of mice, and I one day saw a small snake killed and carried away. It is said that snakes are their common prey during the daytime. I may here mention, as showing on what various kinds of food owls subsist, that a species killed among the islets of the Chonos Archipelago, had its stomach full of good-sized crabs. In India* there is a fishing genus of owls, which likewise catches crabs. http://darwin-online.org.uk/content/frameset?pageseq=1&itemID=F14&viewtype=text
55. In shoaler water, at the distance of a few miles from the coast, very many kinds of crustacea and some other animals are numerous, but only during the night. http://darwin-online.org.uk/content/frameset?pageseq=1&itemID=F14&viewtype=text
56. In the daytime they either lie among the aquatic plants, or openly feed on the turf plain. http://darwin-online.org.uk/content/frameset?pageseq=1&itemID=F14&viewtype=text
57. The four species have nearly similar habits; the peludo, however, is nocturnal, while the others wander by day over the open plains, feeding on beetles, larvæ, roots, and even small snakes. http://darwin-online.org.uk/content/frameset?pageseq=1&itemID=F14&viewtype=text
58. Perhaps they may vary in colour according to the period of the year. http://darwin-online.org.uk/content/frameset?pageseq=1&itemID=F8.16&viewtype=text
59. This is evidently done for their sport; or, perhaps, is connected (for a similar habit may sometimes be observed during the breeding season amongst our common rooks) with their

matrimonial alliances. http://darwin-online.org.uk/content/frameset?pageseq=1&itemID=F9.3&viewtype=text

60. . I believe this bird does not migrate from the Falkland Islands; it builds on the small outlying islets. This latter circumstance is supposed to be owing to the fear of the foxes; and it is perhaps from the same cause, that although very tame by day, they are much the contrary in the dusk of the evening. http://darwin-online.org.uk/content/frameset?pageseq=1&itemID=F9.3&viewtype=text
61. This elegant bird, which is conspicuous amongst most land species by the whiteness of its plumage, is found, though not commonly, (in November) in Banda Oriental; whilst near Santa Fé, three degrees of latitude northward, it was common during the same time of year. http://darwin-online.org.uk/content/frameset?pageseq=1&itemID=F9.3&viewtype=text
62. In the breeding season it flies upward, and then falls to the ground, with raised wings, in the peculiar manner common to the Anthus arboreus of England. [...] his bird is commonly called the jackass penguin, from its habit, while on shore, of throwing its head backwards, and making a loud strange noise, very like the braying of that animal; but while at sea, and undisturbed, its note is very deep and solemn, and is often heard in the night-time. http://darwin-online.org.uk/content/frameset?pageseq=1&itemID=F10.3&viewtype=text
63. At the time of year I refer to, there were very few flowers, and none whatever near the beds of bromelia. Hence I was quite sure they did not live on honey; and on opening the stomach and upper intestine, by the aid of a lens I could plainly distinguish, in a yellow fluid, morsels of the wings of diptera—probably tipulidæ. It is evident that these birds search for minute insects in their winter-quarters under the thick foliage. I opened the stomachs of several specimens, which were shot in different parts of the continent; and in all, remains of insects were so numerous, as often to present a black comminuted mass, as in the stomach of a creeper. In central Chile these birds are migratory: they make their appearance there in autumn, and in the latter end of the month corresponding to our October, they were very common. In the spring they began to disappear, and on the 12th of what would correspond to our March, in the course of a long walk, I saw only one individual. As this species migrates to the southward, it is replaced by the arrival of a larger kind, which will be presently described. I do not believe the small kind breeds in Chile; for, during the summer, their nests were common to the south of that country. The migration of the humming-birds on both the east* and west coast of North America exactly corresponds to what takes place in this southern continent. In both cases they move towards the tropic during the colder parts of the year, and retreat northward before the returning heat. Some, however, remain during the whole year in Tierra del Fuego; and in Northern California,—which in the northern hemisphere has the same relative position which Tierra del Fuego has in the southern,—some, according to Beechey, likewise remain. http://darwin-online.org.uk/content/frameset?pageseq=1&itemID=F10.3&viewtype=text
64. If it is an unnamed species, surely it ought to be called diabolicus, for it is a fit toad to preach in the ear of Eve. Instead of being nocturnal in its habits, as other toads are, and living in damp obscure recesses, it crawls during the heat of the day about the dry sand-hillocks and arid plains, where not a single drop of water can be found. It must necessarily depend on the dew for its moisture; and this probably is absorbed by the skin, for it is known, that these reptiles possess great powers of cutaneous absorption. At Maldonado, I found one in a situation nearly as dry as at Bahia Blanca, and thinking to give it a great treat, carried it to a pool of water; not only was the little animal unable to swim, but, I think, without help would soon have been drowned. http://darwin-online.org.uk/content/frameset?pageseq=1&itemID=F10.3&viewtype=text
65. I may mention as a proof of how great a difference there is between the seasons of the wooded and the open parts of the coast, that on September 20th, in lat. 34°, these birds had young ones in the nest, while among the Chonos Islands, three months later in the summ,

they were only laying; the difference in latitude between these two places being about 700 miles. http://darwin-online.org.uk/content/frameset?pageseq=1&itemID=F10.3&viewtype=text

66. Daily, in the neighbourhood of these spots, the Cavies were abundant: but differently from most burrowing animals, they wander, commonly two or three together, to miles or leagues from their home; nor do I know whether they return at night. The Cavy feeds and roams about by day. http://darwin-online.org.uk/content/frameset?pageseq=1&itemID=F8.10&viewtype=text
67. All the species, excepting one, wander about by day. http://darwin-online.org.uk/content/frameset?pageseq=1&itemID=F8.10&viewtype=text
68. At certain seasons it frequently utters a peculiar, shrill but gentle, reiterated cry, which is so quickly repeated as to produce one running sound. http://darwin-online.org.uk/content/frameset?pageseq=1&itemID=F8.11&viewtype=text
69. They feed by day in a fearless manner. http://darwin-online.org.uk/content/frameset?pageseq=1&itemID=F8.10&viewtype=text
70. In the breeding season it flies upward, and then falls to the ground, with raised wings, in the peculiar manner common to the Anthus arboreus of England. http://darwin-online.org.uk/content/frameset?pageseq=1&itemID=F8.11&viewtype=text
71. It is very tame, and commonly feeds by day: it is said to bring forth two young ones at a birth. http://darwin-online.org.uk/content/frameset?pageseq=1&itemID=F8.10&viewtype=text
72. At this time of the year, many of the does had just kidded. I was informed, by the Spaniards, that this deer sheds its horns every year. http://darwin-online.org.uk/content/frameset?pageseq=1&itemID=F9.2&viewtype=text
73. At Maldonado they often may be seen during the day, seated on the grassy plain in small groups of three http://darwin-online.org.uk/content/frameset?pageseq=1&itemID=F9.2&viewtype=text
74. During the breeding season, when the male and female are together, the male utters a hoarse roar or bellowing, which it is said, can be heard at the distance of more than a hundred yards. The female never uses her voice, and the male only at such times. http://darwin-online.org.uk/content/frameset?pageseq=1&itemID=F10.3&viewtype=text
75. The Birgos is diurnal in its habits, but every night it is said to pay a visit to the sea, no doubt for the purpose of moistening its branchiæ. http://darwin-online.org.uk/content/frameset?pageseq=1&itemID=F10.3&viewtype=text
76. In Patagonia, the condors either by pairs or many together, both sleep and breed on the same overhanging ledges. In Chile, during the greater part of the year, they haunt the lower country near the shores of the Pacific, and at night several roost in one tree; but in the early part of summer, they retire to the most inaccessible parts of the inner Cordillera, there to breed in peace. With respect to their propagation, I was told by the country people in Chile, that the condor makes no sort of nest, but in the months of November and December lays two large white eggs on a shelf of bare rock. On the Patagonian coast I could not see any sort of nest among the cliffs, where the young ones were standing. It is said the young condors cannot fly for an entire year. At Concepcion, on the fifth of March (corresponding to our September), I saw a young bird, which, though in size little inferior to an old one, was completely covered by down like that of a gosling, but of a blackish colour. I feel sure this bird could not have used its wings for flight for many months. After the period when the young condors can fly, and apparently as well as the old birds, they yet remain both roosting at night on the same ledge, and hunting by day with their parents. Before, however, the young bird has the ruff round its neck turned white, it may often be seen hunting by itself. At the mouth of the St. Cruz, during part of April and May, a pair of old birds might be seen every day either perched on a certain ledge, or sailing about in company with a single young one, which latter though full fledged, had not its ruff white. I should think, especially when recollecting the state in which the Concepcion bird was on the previous month, that this young condor had not been hatched from an egg of that summer. As there were no other young birds, it seems probable that the

condor only lays once in two years.http://darwin-online.org.uk/content/frameset?pageseq=1&itemID=F10.3&viewtype=text

77. Edward Blyth, "Observations on the various seasonal and other external changes which regularly take place in Birds, more particularly in those which occur in Britain ; with Remarks on their great Importance in indicating the true Affinities of Species; and upon the Natural System of Arrangement ", Magazine of Natural History, vol. 9, 1836 pp. 393-409; on p. 407: "[referring to those who] hold that every natural assemblage of species, great or small, forms part of some quinary circle. Now, I cannot but observe here … I should think that a due consideration of this first binary distribution must at once carry conviction of the mind, must be at once a most unanswerable argument against all quinary or similar doctrines …". http://darwin-online.org.uk/content/frameset?pageseq=1&itemID=CUL-DAR122.-&viewtype=text
78. It was not the flowering season, but even at this time of the year there were some very pretty oxalises and mesembryanthemums, and on the sandy spots fine tufts of heaths. http://darwin-online.org.uk/content/frameset?pageseq=1&itemID=F10.3&viewtype=text
79. "This species appeared to be common at Pernambuco (five degrees north of Bahia). Upon entering an old lime-kiln in the middle of the day, I disturbed a considerable number of them: they did not seem to be much incommoded by the light, and their habitation was much less dark than that usually frequented as a sleeping place by these animals." http://darwin-online.org.uk/content/frameset?pageseq=1&itemID=F8.2&viewtype=text
80. In the middle of the day, they frequently roll in the dust, in saucer-shaped hollows. http://darwin-online.org.uk/content/frameset?pageseq=1&itemID=F8.4&viewtype=text
81. Of this species, there are six specimens in Mr. Darwin's collection ; two were found "living in the short grass, near the summit of the Island of Ascension, where the climate is temperate."— D. Two others were procured "on a small, stony, and arid island, near Porto Praya, the capital of St. Jago, in the Cape de Verde Islands,—climate very hot and dry. Excepting during the rainy season, which is of short duration, these little animals can never taste fresh water, nor does the island afford any succulent plant." http://darwin-online.org.uk/content/frameset?pageseq=1&itemID=F8.5&viewtype=text
82. These willings have relation to external contingencies, as much as growth of tissue and are subject to accident; the sexual willing comes on period of year as much as inflorescence.— *
83. * Has any vegetable or animal matter been formed by the union of simple non-organic matter without action of vital laws. http://darwin-online.org.uk/content/frameset?pageseq=1&itemID=CUL-DAR91.4-55&viewtype=text
84. Of migrations of birds he mentioned many most curious cases. The birds seem to follow narrow bands, certain kinds as gallinules taking the low country near coast & other the mountains, & then5 | appearing to remain about a fortnight. See Silliman's Journal 1837. Paper by Bachman.6 that is succession of birds. — in some species as Tanagra males come first & then females in flocks as in English nightingales — other birds (& this seems common kind migration of America) migrate singly flying few miles every day & generally by night — other birds which is strictly diurnal, migrates singly by night. — others in flocks. These birds seem clearly directed by kind of country; kind of migration quite different in species of same genus. The Muscicapa solitaria stay about a fortnight in one particular part of country, like White7 of Selborne Rock Ouzels. If the line of bands or country (These facts show the normal condition of migration)8 http://darwin-online.org.uk/content/frameset?pageseq=1&itemID=F1574e&viewtype=text
85. — Mr Yarrell1 says the male Axis of India, breeds at times when horns not perfect — (is not this so in S. America with C. campestris, refer to my notes) & Mr Yarrell supposes this a consequence of that female breeding all the year round. Ask Colonel Sykes2. Even our domesticated cattle have tendency to breed at particular times. http://darwin-online.org.uk/content/frameset?pageseq=1&itemID=F1574c&viewtype=text

86. says some of the species of smaller petrels are night birds agree with [word deleted] nocturnal habits of Crustacea. http://darwin-online.org.uk/content/frameset?pageseq=1&itemID=CUL-DAR118.-&viewtype=text
87. The continuity of the discussion on the Ostrich given in the above text, was only reached after one major deletion, and lesser corrections. I give here the text of the long deletion, to be replaced by (a), p. 82 (a). "now although it is probable from what I have seen & heard of the state of the Ovarium of the Hen, & from the number of eggs found in one district so [very del.] extraordinarily large in proportion to that of the parent birds [query owing to blot] that she may lay that number in the season, yet the time she must require is very long. — Before the last egg was laid the first would probably be addled [decayed del.] — If we believe that all the [each del.] females lay each a few eggs in several nests; the eggs in such nests, might be collected within a short period. — We shall thus explain the extraordinary number of eggs found in any district; and moreover | each cock bird some time during the whole season of laying [incubation del.] will be employed in incubation; and at a period, when the greater number of females could not sit, owing to not having finished laying. —. http://darwin-online.org.uk/content/frameset?pageseq=1&itemID=F1577&viewtype=text
88. In the evening this bird seats itself on a branch & continually repeats, without any alteration, a shrill & rather agreeable cry, which somewhat resembles articulate words. The Spaniards, say it is like the words 'Bien te veo' (I see you well). & accordingly have given this name to this Bird. — http://darwin-online.org.uk/content/frameset?pageseq=1&itemID=F1577&viewtype=text
89. Although flying all day on a moonlight night they may be still seen on the wing. — I was told by a Sealer that they, together with (1335) & Mother Cary's Chicken, all build in the cliffs of South Georgia; And that no other breeding place is known of. — They all arrive very regularly in September & leave again in the Autumn. — That the Albatross alone stay the Winter. — http://darwin-online.org.uk/content/frameset?pageseq=1&itemID=F1577&viewtype=text
90. — The second method is to mark the trees in which they roost, frequently to the number of five or six together, [corrected from: in which the Condors frequently to the number of five or six together, roost,] & then at night climb up & noose them; they are such heavy sleepers, as I have myself witnessed. http://darwin-online.org.uk/content/frameset?pageseq=1&itemID=F1577&viewtype=text
91. They do not migrate, but build on the small outlying islets; this is supposed to be from fear of the Foxes, & it is perhaps from the same cause, that these geese, though exceedingly tame by day, are shy & wild in the dusk of the evening. http://darwin-online.org.uk/content/frameset?pageseq=1&itemID=F1577&viewtype=text
92. There were at this time of year scarcely any flowers, & none whatever, where the above plants grew. Hence I was well assured they did not live on honey; on opening the stomach or duodenum, in a yellow fluid, by the help of a lens, I plainly saw numerous morcels of the wings of Diptera, probably Tipulidae. http://darwin-online.org.uk/content/frameset?pageseq=1&itemID=F1577&viewtype=text
93. It is moreover an active bird & generally making a noise; these noises are various & strangely odd, some, are like cooing of doves, others like bubbling water, & many defy all similes. The country people, say it changes its cry, five times in the year; so that I suppose, they vary them according to the Season. http://darwin-online.org.uk/content/frameset?pageseq=1&itemID=F1577&viewtype=text
94. When at sea, undisturbed, its note is very deep solemn, is often heard in the night time. http://darwin-online.org.uk/content/frameset?pageseq=1&itemID=F1577&viewtype=text
95. Owl. —Excessively numerous, mentioned by all travellers as one of the most striking features in the | ornithology of the Pampas. They live in burrows, which they excavate, on any level sandy part; but on the Buenos Ayres side of the Plata, where the Biscatche are found, they appear exclusively to use the holes of that animal. During the open day, but more especially

in the evening, these birds may be seen in every direction, standing, frequently by pairs, on the hillock, by their burrows. Whence they quietly gaze on the passer by; if disturbed, they either enter the hole, or, uttering a shrill harsh cry move with a remarkably undulatory flight to a short distance; whence again they gaze at their pursuer. Occassionally, in the evening they may be heard hooting. I found in their stomachs, the remains of mice; & I one day saw a small snake, killed & carried away. It is said, that these latter animals, are their chief object of prey during the day. If, by the means of the traps, I had not been aware how wonderfully numerous the smaller rodentia are in these open countries, it would have been an enigma to explain the support of such an infinity of owls. http://darwin-online.org.uk/content/frameset?keywords=one%20most%20travellers%20of%20in%20excessively%20by%20numerous%20the%20all%20mentioned%20features%20as%20striking&pageseq=25&itemID=F1577&viewtype=text

96. Caprimulgus, male: utters at night a simple gentle, plaintive cry, which is regarded with much superstitious dread by the natives; frequents the hills. http://darwin-online.org.uk/content/frameset?pageseq=1&itemID=F1577&viewtype=text
97. The little owl (Noctua cunicularia), which has been so often mentioned, on the plains of Buenos Ayres exclusively inhabits the holes of the bizcacha; but in Banda Oriental it is its own workman. During the open day, but more especially in the evening, these birds may be seen in every direction standing frequently by pairs on the hillock near their burrows. If disturbed they either enter the hole, or, uttering a shrill harsh cry, move with a remarkably undulatory flight to a short distance, and then turning round, steadily gaze at their pursuer. Occasionally in the evening they may be heard hooting. I found in the stomachs of two which I opened the remains of mice, and I one day saw a small snake killed and carried away. It is said these latter animals are their common prey during the daytime. I may here mention, as showing on what various kinds of food owls subsist, that a species that was killed among the islets of the Chonos Archipelago, had its stomach full of good-sized crabs. http://darwin-online.org.uk/content/frameset?pageseq=1&itemID=F10.3&viewtype=text
98. Every leaf (excepting those on the surface) is white with such Corallines or Corallinas & Spirobæ1 & compound Ascidiæ2. Examining these with strong microscope, infinite |243| numbers of minute Crustaceæ will be seen.— The number of compound & simple Ascidiæ is a very observable fact.— as in a lesser degree are the Holuthuriæ & Asterias.— [note (b)] The number of Corallinas inarticulæ, encrusting & coating rocks & shells both in & out of Tidal influence is very observable.— [note ends] On shaking the great entangled roots it is curious to see the heap of fish, shells, crabs, sea-eggs, Cuttle fish, star fish, Planariæ, Nereidæ3, which fall out.— This latter tribe I have much neglected.— Amongst the Gasteropoda, Pleurobranchus4 is common: but Trochus5 & patelliform shells abound on all the leaves.— One single plant form is an immense & most interesting menagerie.— If this Fucus was to cease living, with it would go many: the Seals, the Cormorants & certainly the small fish & then sooner or later the Fuegian man must follow.— the greater number of the invertebrates would likewise perish, but how many it is hard to conjecture. http://darwin-online.org.uk/content/frameset?pageseq=1&itemID=F1840&viewtype=text
99. Followed by deleted words: 'the foliage is scanty & of a rather peculiar light green tint; it is not periodically shed'. http://darwin-online.org.uk/content/frameset?pageseq=1&itemID=F1925&viewtype=text
100. The animal was slightly phosphorescent at night. http://darwin-online.org.uk/content/frameset?pageseq=1&itemID=F1840&viewtype=text
101. There are at this time of year scarcely any flowers, & none whatever where the above plants grow. http://darwin-online.org.uk/content/frameset?pageseq=1&itemID=F1840&viewtype=text
102. In the evening this bird seats itself on a branch & repeats continually a shrill rather agreeable note without any alteration. http://darwin-online.org.uk/content/frameset?pageseq=1&itemID=F1840&viewtype=text

103. (a) At this time of year (end of September & beginning of October) the females have numerous large elongated eggs.— These they lay in their burrows & the inhabitants seek for them to eat. http://darwin-online.org.uk/content/frameset?pageseq=1&itemID=F1840&viewtype=text
104. Is it possible that the terminal buds are periodically shedded? http://darwin-online.org.uk/content/frameset?pageseq=1&itemID=F1840&viewtype=text
105. Scarabeidæ14 not abundant (owing I suppose to season). http://darwin-online.org.uk/content/frameset?pageseq=1&itemID=F1840&viewtype=text
106. The Peludo is a nocturnal animal, & is caught by people going out at night with dogs. — This is the only species which is found in the vicinity of B. Ayres. http://darwin-online.org.uk/content/frameset?pageseq=1&itemID=CUL-DAR29.1.A1-A49&viewtype=text
107. [Note] (a): I once saw this animal sitting on a lofty pinnacle (probably 8000 ft above sea) in the Pass of the Patillo. It inhabits alone very lofty places: I have seen it at a height of about 6000 ft. Lives under the stones; comes out in the evening, at which time & during the early part of the night utters a low whistle. — In this respect differs from the Biscatche of the plains. — At the distance appeared to be more reddish in the breast; & tail with larger brush to it. http://darwin-online.org.uk/content/frameset?pageseq=1&itemID=CUL-DAR29.1.A1-A49&viewtype=text
108. Didelphis: inhabits burrows, nocturnal in its habits;. http://darwin-online.org.uk/content/frameset?pageseq=1&itemID=CUL-DAR29.1.A1-A49&viewtype=text
109. On gloomy days & in the evening they come out to feed; are not very timid & can easily be shot. http://darwin-online.org.uk/content/frameset?pageseq=1&itemID=CUL-DAR29.1.A1-A49&viewtype=text
110. Pichey Mataco, Peluda1 all inhabit same plain, first wonderfully abundant, buries itself with great celerity: has 2 or 3 young at one time. Molina does not go South of Tandil. Taupes different note here, single, repeated at equal times, or accelerating, very noisy in evening after sunset, quiet at night. http://darwin-online.org.uk/content/frameset?pageseq=1&itemID=F1571&viewtype=text
111. Ostriches lay eggs in middle of day. http://darwin-online.org.uk/content/frameset?keywords=middle%20eggs%20of%20in%20ostriches%20day%20lay&pageseq=63&itemID=EH88202331&viewtype=text
112. Yearly the Polypus has to replace this damage. http://darwin-online.org.uk/content/frameset?pageseq=1&itemID=F1576&viewtype=text
113. Although this is true, yet there is a charm in the unconfined feeling of walking over the boundless turf plain: Moreover if your view is limited to a small space, many objects possess great beauty.— Some of the smallest birds are most brilliantly coloured; much more so than those in Brazil.— The bright green turf being browsed short by the cattle, is ornamented by dwarf flowers; amongst which to my eyes the Daisy claimed the place of an old friend.— The only other plants of larger size are tall rushes & a thistle resembling much the Acanthus; this latter with its silvery foliage covers large spaces of ground.— I went on board with a party of midshipmen; who had been shooting & had killed several brace of Partridges & wild Ducks, & had caught a large Guano about 3 feet long.— These lizards at certain times of the year are reckoned excellent food.— The evening was calm & bright, but in the middle of night it blew a sudden gale.— All hands were piped up to send Top-gallant masts on deck & to get in the Cutter: In such scenes of confusion, I am doubtful whether the war of the elements or shouts of the officers be most discordant.— http://darwin-online.org.uk/content/frameset?pageseq=1&itemID=F1925&viewtype=text
114. Pasture for animals at this time of year could not be procured. http://darwin-online.org.uk/content/frameset?pageseq=1&itemID=F1925&viewtype=text

115. Nature in these Zones chooses her vocalists out of other tribes; in the evening some species of frogs make a concert no ways unpleasant. this as the night advances is accompanied by the endless cry of the Cicadas.— As far as regards insects, M. Lacordaire1 states the months during which I have collected are by no means the most productive in insects.— This may account for the few numbers of large & brilliant beetles which I have seen.— Of the smaller species I certainly have succeeded in taking great numbers.— http://darwin-online.org.uk/content/frameset?pageseq=1&itemID=F1840&viewtype=text

116. From finding Cassida, Crysomela, Curculionidous, Heteromerous, Lamellicorns, Carabidous beetles, & Epeira amongst spiders, under stones: from Vaginulus2 & land shells with a membrane over the mouth being in same site; from finding Bufo3 & Lacerta4 half torpid; it is clear animals are now hybernating.— Considering the high temperature, this is curious.— From 276 observ: made at 2 hours intervals during 23 days from July 27 to August 19th (both inclusive), mean temp is 58°.4.— Mean hottest day 65°.5 do. Coldest day 45°.8 The lowest point the Thermometer fell to was 41°.5; it occasionally in middle of the day rose to 69° or 70°.— http://darwin-online.org.uk/content/frameset?pageseq=1&itemID=F1840&viewtype=text

117. C X Crustaceæ. pelagic. Watchman Cape. L. 48°18′ [notes opposite] Caught at night, could not catch any by day under similar circumstances: small white Entom. creeping numerous. Small white Ento: with long antennæ very numerous at night Lat 51°53′ Long. 68°11′— * http://darwin-online.org.uk/content/frameset?pageseq=1&itemID=F1840&viewtype=text

118. They are torpid slow animals, crawling when not frightened with their belly & tail on the ground.— frequently they doze on the parched ground, with their eyes closed & hind legs stretched outwards.— In none of their motions, is there that celerity & alertness which is so conspicuous in true Lacertas & Iguanas.— Their habits are diurnal: they seldom leave wander to any distance from their burrows: when frightened they rush to them with a most awkward gait: excepting going down hill their motion, from the lateral position of their legs, is not quick.— They are not timorous. When attentively watching an intruder they curl their tails, & raising themselves as if in defiance on their front legs, vertically shake their heads with a quick motion.— I have seen small Muscivorous Lizards perform the same gestures.— This gives them rather a fierce aspect, but in truth they are far the contrary. When however |338| being caught & plagued with a stick they will bite it severely.— Two being placed on the ground close together will fight & bite each other till blood is drawn. http://darwin-online.org.uk/content/frameset?pageseq=1&itemID=F1840&viewtype=text

119. These birds are common far inland near the R. Parana. They rest on the grass plains, in same manner as in day time near the sea on mud banks: are said to stay whole year & breed in the marshes. One evening near Rozario, as it was growing dark, we were anchored in a narrow Riacho or arm; here there were many smaller fry, & I saw one of these birds rapidly flying up & down ploughing the water as described at Maldonado. Class. Dic. is aware of this habit.— I think these & other marine birds perhaps enter far inland the more nearly from its extreme flatness. [note ends] [the Scissor-beak Rhynchops nigra Linn. is discussed in Zoology 3:143-4, and it is mentioned that Richard Owen had dissected the head of a specimen brought home by CD in spirits, but had not found any special innervation in the lower mandible. See also Ornithological Notes pp. 221-3] http://darwin-online.org.uk/content/frameset?pageseq=1&itemID=F1840&viewtype=text

120. At Monte Video I observed that some large flocks during the day remained on the mud-banks at the head of the harbour, in the same manner as on the grassy plains near the Parana; and every evening they took flight direct to seaward. From these facts, I suspect that the Rhyncops generally fishes by night, at which time many of the lower animals come most abundantly to the surface. http://darwin-online.org.uk/content/frameset?pageseq=1&itemID=F10.3&viewtype=text

121. The Paluda is a nocturnal animal & is taken by going out at night with dogs http://darwin-online.org.uk/content/frameset?pageseq=1&itemID=F1840&viewtype=text
122. at this time of year nothing but an amphibious animal could tolerate the climate. http://darwin-online.org.uk/content/frameset?pageseq=1&itemID=F1925&viewtype=text
123. This animal is nocturnal in its habits; http://darwin-online.org.uk/content/frameset?keywords=is%20habits%20in%20animal%20its%20nocturnal%20this&pageseq=147&itemID=F1925&viewtype=text

Time as a bias

124. I have examined a great number of specimens from various localities, taken at different times of the year. http://darwin-online.org.uk/content/frameset?keywords=at%20examined%20of%20great%20a%20from%20have%20year%20number%20the%20times%20i%20various%20specimens%20taken%20localities%20different&pageseq=257&itemID=F339.1&viewtype=text
125. Although I have dissected, at least, thirty specimens, taken at different times of the year, and from different localities, and when many of the specimens were mature and ready for the impregnation of ova, as clearly shown by the presence of innumerable spermatozoa, I have never seen even a trace of an ovum or ovaria. http://darwin-online.org.uk/content/frameset?keywords=at%20examined%20of%20great%20a%20from%20have%20year%20number%20the%20times%20i%20various%20specimens%20taken%20localities%20different&pageseq=257&itemID=F339.1&viewtype=text
126. As soon as many flowers were open, I began to examine them for twenty-three consecutive days: I looked at them after hot sunshine, after rain, and at all hours. https://darwin-online.org.uk/content/frameset?itemID=F800&keywords=i%20for%20days%20three%20began%20examine%20them%20twenty%20consecutive%20to&viewtype=text&pageseq=63
127. Enamel and Dentine.—As the secretion decalcified ordinary bone, I determined to try whether it would act on enamel and dentine, but did not expect that it would succeed with so hard a substance as enamel. Dr. Klein gave me some thin transverse slices of the canine tooth of a dog; small angular fragments of which were placed on four leaves; and these were examined each succeeding day at the same hour. The results are, I think, worth giving in detail. https://darwin-online.org.uk/content/frameset?pageseq=1&itemID=F1249&viewtype=text
128. Now it is highly desirable that it should be ascertained whether or not these flowers are visited at night by any of the innumerable individuals of the many species of minute moths. A lepidopterist while collecting at night, if endowed with only a small portion of the indomitable patience displayed by Müller, could ascertain this fact. http://darwin-online.org.uk/content/frameset?keywords=at%20night%20while%20a%20collecting%20lepidopterist&pageseq=10&itemID=F1432&viewtype=text
129. There is, however, some liability to error in forming a judgment on this head, from the extreme difficulty of ascertaining whether flowers which are rarely or never visited during the day (as in the above given case of Fumaria capreolata) are not visited by small nocturnal Lepidoptera, which are known to be strongly attracted by sugar. http://darwin-online.org.uk/content/frameset?pageseq=1&itemID=F1249&viewtype=text
130. Many species which bear small and inconspicuous flowers are never, or most rarely, visited by insects during the day; and Hermann Müller infers that they must be always, or almost always, self-fertilised. But the evidence appears to me insufficient, until it can be shown that such flowers are not visited during the night by any of the innumerable kinds of small moths. From the simple fact of these small flowers expanding, and from some of them secreting nectar, it seems probable that they are at least occasionally visited and intercrossed by nocturnal insects. http://darwin-online.org.uk/content/frameset?pageseq=1&itemID=F1251&viewtype=text

131. When, however, it is believed on fairly good evidence that the flowers on a plant in its native country do not open at any hour of the day or night, and yet set seeds capable of germination, these may fairly be considered as cleistogamic, notwithstanding that they present no peculiarities of structure. http://darwin-online.org.uk/content/frameset?pageseq=1&itemID=F1281&viewtype=text
132. Shell Rain in the Isle of Wight — I earnestly hope that "C." of Winchester will give some more particulars regarding the fall of shells at Osborne: Were any of the shells living? Over how wide an area did they fall? During how long a time are they believed to have fallen? At what hour and on what day? Did only one kind of shell fall? https://darwin-online.org.uk/content/frameset?itemID=F1685&keywords=of%20shell%20isle%20wight%20rain&viewtype=text&pageseq=1

3. Biological rhythms are variable in populations

1. The habitual "periods" of different families of the same species differ, for instance, in the time of year of reproduction, and the period of life when the capacity is acquired, and the hour of roosting (in Malay fowls), &c., &c. These periodical habits are perhaps essentially corporeal, and may be compared to nearly similar habits in plants, which are known to vary extremely. http://darwin-online.org.uk/content/frameset?keywords=is%20acquired%20and%20time%20of%20reproduction%20in%20year%20life%20the%20capacity%20period%20when&pageseq=149&itemID=F1556&viewtype=text
2. The period of gestation and of maturity, as shown in the earlier chapters,—the season and the frequency of the act of breeding,—have all been greatly modified under domestication. http://darwin-online.org.uk/content/frameset?keywords=and%20of%20earlier%20in%20maturity%20chapters%20gestation%20the%20shown%20period%20as&pageseq=319&itemID=F877.2&viewtype=text
3. The period of flowering has been considerably hastened, and this has probably been effected by continued selection. http://darwin-online.org.uk/content/frameset?keywords=and%20of%20considerably%20by%20hastened%20the%20effected%20selection%20been%20flowering%20period%20probably%20has%20this%20continued&pageseq=385&itemID=F878.1&viewtype=text
4. According to Kölreuter, hybrids in the genus Mirabilis vary almost infinitely, and he describes new and singular characters in the form of the seeds, in the colour of the anthers, in the cotyledons being of immense size, in new and highly peculiar odours, in the flowers expanding early in the season, and in their closing at night. With respect to one lot of these hybrids, he remarks that they presented characters exactly the reverse of what might have been expected from their parentage**.** http://darwin-online.org.uk/content/frameset?keywords=the%20to%20genus%20in%20mirabilis%20according%20k%C3%B6lreuter%20hybrids&pageseq=270&itemID=F880.2&viewtype=text
5. I shall be in London next week, and I will call on you on Thursday morning for one hour precisely, so as not to lose much of your time and my own; but will you let me this time come as early as 9 o'clock, for I have much which I must do in the morning in my strongest time? http://darwin-online.org.uk/content/frameset?keywords=to%20and%20of%20time%20my%20lose%20not%20your%20much%20own&pageseq=143&itemID=F1452.2&viewtype=text
6. He rose early, chiefly because he could not lie in bed, and I think he would have liked to get up earlier than he did. … After breakfasting alone about 7.45, he went to work at once, considering the 1 1/2 hour between 8 and 9.30 one of his best working times. http://darwin-online.org.uk/content/frameset?keywords=early%20in%20bed%20he%20because%20chiefly%20rose%20could%20lie%20not&pageseq=130&itemID=F1452.1&viewtype=text

7. The early morning was the only time at which he could make any effort of the kind, with comparative impunity. Thus it came about that the visits he paid to his scientific friends in London were by preference made as early as ten in the morning. For the same reason he started on his journeys by the earliest possible train, and used to arrive at the houses of relatives in London when they were beginning their day. http://darwin-online.org.uk/content/frameset?keywords=early%20in%20bed%20he%20because%20chiefly%20rose%20could%20lie%20not&pageseq=130&itemID=F1452.1&viewtype=text

4. The Adaptive Value of Biological Rhythms

Sleep movements

1. From the several cases above given, there can be no doubt that the position of the leaves at night affects their temperature through radiation to such a degree, that when exposed to a clear sky during a frost, it is a question of life and death. We may therefore admit as highly probable, seeing that their nocturnal position is so well adapted to lessen radiation, that the object gained by their often complicated sleep movements, is to lessen the degree to which they are chilled at night. http://darwin-online.org.uk/content/frameset?keywords=doubt%20of%20from%20the%20leaves%20cases%20that%20above%20be%20no%20can%20position%20several%20given%20there&pageseq=311&itemID=F1325&viewtype=text
2. It is therefore probable that this sleep-like movement, which occurs only when the ground is dry, is an adaptation against the loss of moisture http://darwin-online.org.uk/content/frameset?keywords=is%20movement%20that%20probable%20therefore%20sleep%20like%20this%20it&pageseq=365&itemID=F1325&viewtype=text
3. The closure of the flowers will also exclude nocturnal insects which may be ill-adapted for their fertilisation, and the well-adapted kinds at periods when the temperature is not favourable for fertilisation. http://darwin-online.org.uk/content/frameset?keywords=is%20movement%20that%20probable%20therefore%20sleep%20like%20this%20it&pageseq=365&itemID=F1325&viewtype=text
4. The leaves of various plants are said to sleep at night, and it will be seen that their blades then assume a vertical position through modified circumnutation, in order to protect their upper surfaces from being chilled through radiation. http://darwin-online.org.uk/content/frameset?pageseq=1&itemID=F1325&viewtype=text
5. In some species the petioles rise up greatly at night, and the pinnae close together. The whole plant is thus rendered more compact, and a much smaller surface is exposed to radiation.
   http://darwin-online.org.uk/content/frameset?pageseq=1&itemID=F1325&viewtype=text
6. Considering that leaves in assuming their nyctitropic positions often move through an angle of 90°; that the movement is rapid in the evening; that in some cases, as we shall see in the next chapter, it is extraordinarily complicated; that with certain seedlings, old enough to bear true leaves, the cotyledons move vertically upwards at night, whilst at the same time the leaflets move vertically downwards; and that in the same genus the leaves or cotyledons of some species move upwards, whilst those of other species move downwards;—from these and other such facts, it is hardly possible to doubt that plants must derive some great advantage from such remarkable powers of movement. http://darwin-online.org.uk/content/frameset?pageseq=1&itemID=F1325&viewtype=text
7. This view that the sleep of leaves saves them from being chilled at night by radiation, would no doubt have occurred to Linnaeus, had the principle of radiation been then discovered; for he suggests in many parts of his 'Somnus Plantarum' that the position of the leaves at night protects the young stems and buds, and often the young inflorescence, against cold winds. We are far from doubting that an additional advantage may be thus gained; and we have observed with several plants, for instance, Desmodium gyrans, that whilst the blade of the

leaf sinks vertically down at night, the petiole rises, so that the blade has to move through a greater angle in order to assume its vertical position than would otherwise have been necessary; but with the result that all the leaves on the same plant are crowded together as if for mutual protection. http://darwin-online.org.uk/content/frameset?pageseq=1&itemID=F1325&viewtype=text

Other traits

8. As, however, plumes would probably be inconvenient and certainly of no use during the winter, it is possible that the habit of moulting twice in the year may have been gradually acquired through natural selection for the sake of casting off inconvenient ornaments during the winter. http://darwin-online.org.uk/content/frameset?keywords=inconvenient%20certainly%20and%20be%20however%20probably%20plumes%20would%20as&pageseq=198&itemID=F937.2&viewtype=text
9. The fact of a larger proportion of white flowers smelling sweetly may depend in part on those which are fertilised by moths requiring the double aid of conspicuousness in the dusk and of odour. Most flowers which are fertilised by crepuscular or nocturnal insects emit their odour chiefly or exclusively in the evening, and they are thus less likely to be visited and have their nectar stolen by ill-adapted diurnal insects. Some flowers, however, which are highly odoriferous depend solely on this quality for their fertilisation, such as the night-flowering stock (Hesperis) and some species of Daphne; and these present the rare case of flowers which are fertilised by insects being obscurely coloured. http://darwin-online.org.uk/content/frameset?keywords=the%20smelling%20flowers%20larger%20white%20of%20proportion%20a%20fact%20sweetly&pageseq=2&itemID=CUL-DAR209.4.187-189&viewtype=text
10. Changes of instinct may sometimes be facilitated by instances occurring of very the same species having, though the cases are same case, different instincts at different periods of life, or time of the year, or when placed under different circumstances &c; in which case either one instinct or the other being might be preserved by natural selection: and such cases of diversity of instinct in the same species can be shown to occur in nature. http://darwin-online.org.uk/content/frameset?keywords=instances%20instinct%20may%20be%20of%20occurring%20by%20sometimes%20facilitated%20changes&pageseq=1&itemID=NHM-MSS-DARA&viewtype=text
11. The tops of the branches were barely covered by water at the time of lowest tide. Several facts having led me to disbelieve in any elevation of the whole atoll, I was at first unable to imagine what cause could have killed so large a field of coral. Upon reflection, however, it appeared to me that the closing up of the above mentioned channels would be a sufficient cause; for before this, a strong breeze by forcing water through them into the head of the lagoon, would tend to raise its level. But now this cannot happen, and the inhabitants observe that the tide rises to a less height, during a high S.E. wind, at the head than at the mouth of the lagoon. The corals, which, under the former condition of things, had attained the utmost possible limit of upward growth, would thus occasionally be exposed for a short time to the sun, and be killed. http://darwin-online.org.uk/content/frameset?keywords=water%20at%20of%20time%20barely%20by%20tide%20the%20were%20tops%20covered%20branches%20lowest&pageseq=33&itemID=F271&viewtype=text
12. The time of pairing, I believe, always falls at a period when the animal is at full vigour; though no doubt it is of still more consequence that the young should be produced at a time when food is superabundant & the other conditions of life favourable: hence it is in itself highly probable that nearly all animals pair annually or biennially according to the period of gestation. We have seen how great has been the actual increase of horses & cattle, in short periods, though many must have been slaughtered or killed by accidents; & these animals,

when compared to the great mass of living beings must be considered as extremely slow breeders: we know the actual rate of doubling of man, a still slower breeder; & we have seen the possible increase of the supposed slowest breeder, the elephant, if allowed to live & breed at its natural rate, even for a few centuries, whereas we have to consider hundreds of thousands of years. Therefore I consider nothing can be more certain, than that every single species on the face of this earth would rapidly swarm to an incalculable degree, if many individuals were not continually destroyed at some period of their lives from the egg or seed upwards, either during each generation or at short intervals in the successive generations. http://darwin-online.org.uk/content/frameset?keywords=is%20believe%20at%20full%20always%20animal%20a%20vigour%20the%20i%20falls%20period%20when&pageseq=26&itemID=CUL-DAR10.1.%281-78%29&viewtype=text

13. But is this the case with smaller changes? There are persons who can see distinctly only in a dull light, and this condition depends, I believe, on the abnormal sensitiveness of the retina, and is known to be inherited. Now, if a bird, for instance, received some great advantage from seeing well in the twilight, all the individuals with the most sensitive retina would succeed best and be the most likely to survive; and why should not all those which happened to have the eye itself a little larger, or the pupil capable of greater dilatation, be likewise preserved, whether or not these modifications were strictly simultaneous? These individuals would subsequently intercross and blend their respective advantages. By such slight successive changes, the eye of a diurnal bird would be brought into the condition of that of an owl, which has often been advanced as an excellent instance of adaptation (Darwin and Gray 1868). http://darwin-online.org.uk/content/frameset?keywords=persons%20distinctly%20in%20see%20a%20are%20dull%20can%20light%20who%20only%20there&pageseq=237&itemID=F878.2&viewtype=text
14. Hence that male which at that time is in fullest vigour, or best armed with arms or ornaments of its species, will gain in hundreds of generations some small advantage and transmit such characters to its offspring. http://darwin-online.org.uk/content/frameset?keywords=is%20at%20that%20which%20hence%20time%20in%20fullest%20male%20vigour&pageseq=11&itemID=CUL-DAR6.16-50&viewtype=text
15. Either their food is more liable to failure, or they have not sufficient power of wing to search for it over an extensive area, or during some season of the year it becomes very scarce, and less wholesome substitutes have to be found; and thus, though more fertile in offspring, they can never increase beyond the supply of food in the least favourable seasons. Many birds can only exist by migrating, when their food becomes scarce, to regions possessing a milder, or at least a different climate, though, as these migrating birds are seldom excessively abundant, it is evident that the countries they visit are still deficient in a constant and abundant supply of wholesome food. Those whose organization does not permit them to migrate when their food becomes periodically scarce, can never attain a large population. This is probably the reason why woodpeckers are scarce with us, while in the tropics they arc among the most abundant of solitary birds. http://darwin-online.org.uk/content/frameset?keywords=is%20to%20liable%20power%20they%20have%20not%20more%20or%20failure%20sufficient%20food%20their%20either&pageseq=12&itemID=F350&viewtype=text
16. Hence these plants not only have lost their proper dimorphic structure and peculiar functional powers, but have acquired an abnormal grade of fertility—unless, indeed, their high fertility may be accounted for by the stigmas receiving pollen from the circumjacent anthers at exactly the most favourable period. http://darwin-online.org.uk/content/frameset?pageseq=1&itemID=F1742&viewtype=text
17. Formerly it appeared to me probable, that the increased fertility of these dimorphic plants might be accounted for by the stigma lying so close to the anthers that it was impregnated at the most favourable age and time of the day; but this explanation is not applicable to the

above given cases, in which the flowers were artificially fertilised with their own pollen. http://darwin-online.org.uk/content/frameset?pageseq=1&itemID=F1249&viewtype=text

18. As the flowers are visited both by day and night-flying Lepidoptera, I do not think that it is fanciful to believe that the bright-purple tint (whether or not specially developed for this purpose) attracts the day-fliers, and the strong foxy odour the night-fliers. http://darwin-online.org.uk/content/frameset?pageseq=1&itemID=F800&viewtype=text
19. I strongly suspect that some well-known laws with respect to the plumage of male and female birds, in comparison with the plumage of the young, can be explained on the view of plumage having been chiefly modified by sexual selection, acting when the birds have come to the breeding age or during the breeding season; the modifications thus produced being inherited at corresponding ages or seasons, either by the males alone, or by the males and females; but I have not space here to enter on this subject. http://darwin-online.org.uk/content/frameset?pageseq=1&itemID=F373&viewtype=text
20. Fritz Müller gives another instance of the want of absolute perfection in the flowers of another member of the Rubiaceæ, namely, Posoqueria fragrans, which is adapted in a most wonderful manner for cross-fertilisation by the agency of moths. (See 'Bot. Zeitung,' 1866, No. 17.) In accordance with the nocturnal habits of these insects, most of the flowers open only during the night; but some open in the day, and the pollen of such flowers is robbed, as Fritz Müller has often seen, by humble-bees and other insects, without any benefit being thus conferred on the plant. http://darwin-online.org.uk/content/frameset?pageseq=1&itemID=F1277&viewtype=text
21. I suspect that the fishing by day at Maldonado, was not a common circumstance, but owing to the multitudes of small fry left by the draining water. If such is the case, we can see, how their nocturnal habits are in | accordance with the method of fishing, which probably depends as much on the sense of touch as on that of sight. Besides fish, it is not improbable that they catch other animals; of which, many. such as Crustaceae come to the surface far more abundantly, during the night than day time. It appears that the whole structure of the bird, its weak bill, with the lower mandible produced & long wings. are evidently adapted for such habits, & not, as according to M. Lesson, to open & feed on [the del.] Mactrae, buried in the sand banks. http://darwin-online.org.uk/content/frameset?pageseq=1&itemID=F1577&viewtype=text
22. Summary and concluding remarks.—From the fore-going discussion on the various laws of inheritance, we learn that characters often or even generally tend to become developed in the same sex, at the same age, and periodically at the same season of the year, in which they first appeared in the parents. But these laws, from unknown causes, are very liable to change. Hence the successive steps in the modification of a species might readily be transmitted in different ways; some of the steps being transmitted to one sex, and some to both; some to the offspring at one age, and some at all ages. Not only are the laws of inheritance extremely complex, but so are the causes which induce and govern variability. The variations thus caused are preserved and accumulated by sexual selection, which is in itself an extremely complex affair, depending, as it does, on ardour in love, courage, and the rivalry of the males, and on the powers of perception, taste, and will of the female. Sexual selection will also be dominated by natural selection for the general welfare of the species. Hence the manner in which the individuals of either sex or of both sexes are affected through sexual selection cannot fail to be complex in the highest degree. http://darwin-online.org.uk/content/frameset?pageseq=1&itemID=F937.1&viewtype=text
23. From the foregoing facts, more especially from neither sex of certain birds changing colour during either annual moult, or changing so slightly that the change can hardly be of any service to them, and from the females of other species moulting twice yet retaining the same colours throughout the year, we may conclude that the habit of moulting twice in the year has not been acquired in order that the male should assume during the breeding-season an ornamental character; but that the double moult, having been originally acquired for some

distinct purpose, has subsequently been taken advantage of in certain cases for gaining a nuptial plumage. http://darwin-online.org.uk/content/frameset?pageseq=1&itemID=F937.1&viewtype=text

24. This species is extraordinarily abundant within the tidal limits round the shores of Great Britain, and apparently of the northern United States. Besides numerous specimens sent to me from very many English localities, the late Mr. W. Thompson, of Belfast, kindly placed in my hands his very large collection; from these materials it appears that B. balanoides is the only tidal species in the northern parts of our island; but in the south and south-west, it is associated with the Chthamalus stellatus and Balanus perforatus. I doubt whether this species ever lives below the lowest tides; the case of a few specimens being mingled with B. improvisus and crenatus, (mentioned under the latter species,) at the bottom of a rudder of a small vessel, about six feet deep, is hardly an exception, for the water would there be troubled and aërated almost as in a breaker; and on this very rudder the upper two or three feet were coated exclusively by the B. balanoides. This species lives on rocks at both the uppermost and lowest limit of the tides; I am informed by Mr. Thompson, that he has seen specimens attached to a spot not covered by water during neap-tides. As a proof of its tenacity of life, Mr. Thompson informs me that he accidentally kept some specimens in a box, in a warm sitting-room, and found them alive seven days afterwards. This same most accurate observer finds, however, that B. balanoides is very susceptible to brackish water; he says, "that having kept some specimens alive for a week in excellent health, the water being changed once in thirty-six hours, they were one day killed instantly by some water, though brought from the same part of the estuary as usual, having been rendered brackish by much rain having lately fallen." I may recall the fact, that B. improvisus lives daily for hours in absolutely fresh running water. http://darwin-online.org.uk/content/frameset?pageseq=1&itemID=F339.2&viewtype=text
25. Mayo (Philosoph. of Living) quotes Whewell as profound because he says length of days adapted to duration of sleep of man!!! whole universe so adapted!!! & not man to Planets. — instance of arrogance!! http://darwin-online.org.uk/content/frameset?keywords=quotes%20whewell%20he%20length%20because%20says%20as%20profound&pageseq=47&itemID=CUL-DAR123.-&viewtype=text

Periodical struggle for existence

26. The war, however, is not constant, but recurrent in a slight degree at short periods, and more severely at occasional more distant periods; and hence its effects are easily overlooked. It is the doctrine of Malthus applied in most cases with tenfold force. As in every climate there are seasons, for each of its inhabitants, of greater and less abundance, so all annually breed; and the moral restraint which in some small degree checks the increase of mankind is entirely lost. http://darwin-online.org.uk/content/frameset?pageseq=1&itemID=F350&viewtype=text
27. It is good thus to try in our imagination to give any form some advantage over another. Probably in no single instance should we know what to do, so as to succeed. It will convince us of our ignorance on the mutual relations of all organic beings; a conviction as necessary, as it seems to be difficult to acquire. All that we can do, is to keep steadily in mind that each organic being is striving to increase at a geometrical ratio; that each at some period of its life, during some season of the year, during each generation or at intervals, has to struggle for life, and to suffer great destruction. When we reflect on this struggle, we may console ourselves with the full belief, that the war of nature is not incessant, that no fear is felt, that death is generally prompt, and that the vigorous, the healthy, and the happy survive and multiply. http://darwin-online.org.uk/content/frameset?pageseq=1&itemID=F373&viewtype=text
28. In the survival of favoured individuals and races, during the constantly-recurrent Struggle for Existence, we see a powerful and ever-acting form of Selection. The struggle for existence inevitably follows from the high geometrical ratio of increase which is common to all organic

beings. This high rate of increase is proved by calculation,—by the rapid increase of many animals and plants during a succession of peculiar seasons, and when naturalised in a new country. More individuals are born than can possibly survive. A grain in the balance may determine which individuals shall live and which shall die,—which variety or species shall increase in number, and which shall decrease, or finally become extinct. As the individuals of the same species come in all respects into the closest competition with each other, the struggle will generally be most severe between them; it will be almost equally severe between the varieties of the same species, and next in severity between the species of the same genus. On the other hand the struggle will often be very severe between beings remote in the scale of nature. The slightest advantage in certain individuals, at any age or during any season, over those with which they come into competition, or better adaptation in however slight a degree to the surrounding physical conditions, will turn the balance. http://darwin-online.org.uk/content/frameset?pageseq=1&itemID=F387&viewtype=text

29. We should always bear in mind that there is a recurrent struggle for life in every organism, and that in every country a destroying agency is always counteracting the geometrical tendency to increase in every species; and yet without our being able to tell with certainty at what period of life, or at what period of the year, the destruction falls the heaviest. Ought we then to expect to trace the steps by which this destroying power, always at work and scarcely perceived by us, becomes increased, and yet if it continues to increase ever so slowly (without the fertility of the species in question be likewise increased) the average number of the individuals of that species must decrease, and become finally lost. http://darwin-online.org.uk/content/frameset?pageseq=1&itemID=F1556&viewtype=text
30. This kind of selection, however, is less rigorous than the other; it does not require the death of the less successful, but gives to them fewer descendants. This struggle falls, moreover, at a time of year when food is generally abundant, and perhaps the effect chiefly produced would be the alteration of sexual characters, and the selection of individual forms, no way related to their power of obtaining food, or of defending themselves from their natural enemies, but of fighting one with another. http://darwin-online.org.uk/content/frameset?pageseq=1&itemID=F1556&viewtype=text
31. It should always be remembered, that in most cases the checks are recurrent yearly in a small, regular degree, and in an extreme degree during unusually cold, hot, dry, or wet years, according to the constitution of the being in question. http://darwin-online.org.uk/content/frameset?pageseq=1&itemID=F350&viewtype=text
32. Natural Selection. De Candolle's war of nature,—seeing contented face of nature,—may be well at first doubted; we see it on borders of perpetual cold2. But considering the enormous geometrical power of increase in every organism and as ⟨?⟩ every country, in ordinary cases ⟨countries⟩ must be stocked to full extent, reflection will show that this is the case. Malthus on man,—in animals no moral [check] restraint ⟨?⟩—they breed in time of year when provision most abundant, or season most favourable, every country has its seasons,—calculate robins,—oscillating from years of destruction3. If proof were wanted let any singular change of climate ⟨occur⟩ here ⟨?⟩, how astoundingly some tribes ⟨?⟩ increase, also introduced animals4, the pressure is always ready,—capacity of alpine plants to endure other climates,—think of endless seeds scattered abroad,—forests regaining their percentage1,—a thousand wedges2 are being forced into the œconomy of nature. This requires much reflection; study Malthus and calculate rates of increase and remember the resistance,—only periodical. The unavoidable effect of this ⟨is⟩ that many of every species are destroyed either in egg or [young or mature (the former state the more common)]. In the course of a thousand generations infinitesimally small differences must inevitably tell3; when unusually cold winter, or hot or dry summer comes, then out of the whole body of individuals of any species, if there be the smallest differences in their structure, habits, instincts [senses], health &c., ⟨it⟩ will on an average tell; as conditions change a rather larger proportion will be preserved: so if the chief check to increase falls on seeds or eggs, so will, in the course of 1000 generations or ten

thousand, those seeds (like one with down to fly4) which fly furthest and get scattered most ultimately rear most plants, and such small differences tend to be hereditary like shades of expression in human countenance. So if one parent ⟨?⟩ fish deposits its egg in infinitesimally different circumstances, as in rather shallower or deeper water &c., it will then ⟨?⟩ tell. Let hares5 increase very slowly from change of climate affecting peculiar plants, and some other ⟨illegible⟩ rabbit decrease in same proportion [let this unsettle organisation of], a canine animal, who formerly derived its chief sustenance by springing on rabbits or running them by scent, must decrease too and might thus readily become exterminated. But if its form varied very slightly, the long legged fleet ones, during a thousand years being selected, and the less fleet rigidly destroyed must, if no law of nature be opposed to it, alter forms. http://darwin-online.org.uk/content/frameset?pageseq=1&itemID=F1555&viewtype=text

33. But if the time has not yet arrived, may it not at some epoch come, when there will be almost as many specific forms as individuals? I think we can clearly see that this would never be the case. Firstly, there would be no apparent benefit in a greater amount of modification than would adapt organic beings to different places in the polity of nature; for although the structure of each organism stands in the most direct & important relation to many other organic beings, and as these latter/26ii/increase in number & diversity of organisation, the conditions of the one will tend to become more & more complex, & its descendants might well profit by a further division of labour; yet all organisms are fundamentally related to the inorganic conditions of the world, which do not tend to become infinitely more varied. Secondly as the amount of life & number of individual beings, whether or not much diversified, also primarily depends on such inorganic con-ditions; if there exist in any country, a vast number of species (although a greater amount of life could be supported) the average number of individuals of each species must be somewhat less than if there were not so many species; & any species, represented by but few individuals, during the fluctuation in number to which all species must be subject from fluctuations in seasons, number of enemies &c, would be extremely liable to total extinction. Moreover, whenever the number of individuals of any species becomes very small, the ill-effects, as I believe, of close inter breeding would come into play. Lastly we have seen in our Chap. IV & shall presently again see, that the amount of variations, & consequently of variation in a right or beneficial direction for natural selection to seize on & preserve, will bear some relation within any given period, to the number of individuals living & liable to variation during such period: consequently when the descendants from any one species have become modified/26 kk/into very many species, without all become numerous in individuals, which [we] see hardly ever to be the case with all the species of the same genus or family, there will be a check amongst the less common species to their further modification: the lesser number of the individuals serving as a regulator or fly-wheel to the increasing rate of further modification, or the production of new specific forms.
http://darwin-online.org.uk/content/frameset?pageseq=1&itemID=F1583&viewtype=text
34. We do not always bear in mind that though food may be now superabundant, it is not so at all seasons of each recurring year. http://darwin-online.org.uk/content/frameset?pageseq=1&itemID=F373&viewtype=text
35. But the struggle will often be very severe between beings most remote in the scale of nature. The slightest advantage in one being, at any age or during any season, over those with which it comes into competition, or better adaptation in however slight a degree to the surrounding physical conditions, will turn the balance. If we look back to an extremely remote epoch, before man had arrived at the dignity of manhood, he would have been guided more by instinct and less by reason than are savages at the present time. Our early semihuman progenitors would not have practised infanticide, for the instincts of the lower animals are never so perverted as to lead them regularly to destroy their own offspring. There would have been no prudential restraint from marriage, and the sexes would have freely united at an early age. Hence the progenitors of man would have tended to increase rapidly, but checks of

some kind, either periodical or constant, must have kept down their numbers, even more severely than with existing savages. What the precise nature of these checks may have been, we cannot say, any more than with most other animals. We know that horses and cattle, which are not highly prolific animals, when first turned loose in South America, increased at an enormous rate. The slowest breeder of all known animals, namely the elephant, would in a few thousand years stock the whole world. The increase of every species of monkey must be checked by some means; but not, as Brehm remarks, by the attacks of beasts of prey. No one will assume that the actual power of reproduction in the wild horses and cattle of America, was at first in any sensible degree increased; or that, as each district became fully stocked, this same power was diminished. No doubt in this case and in all others, many checks concur, and different checks under different circumstances; periodical dearths, depending on unfavourable seasons, being probably the most important of all. So it will have been with the early progenitors of man. http://darwin-online.org.uk/content/frameset?pageseq=1&itemID=F937.1&viewtype=text

36. Climate plays an important part in determining the average numbers of a species, and periodical seasons of extreme cold or drought seem to be the most effective of all checks. http://darwin-online.org.uk/content/frameset?pageseq=1&itemID=F391&viewtype=text
37. If now we have succeeded in establishing these two points—1st, that the animal population of a country is generally stationary, being kept down by a periodical deficiency of food, and other checks; and, 2nd, that the comparative abundance or scarcity of the individuals of the several species is entirely due to their organization and resulting habits, which, rendering it more difficult to procure a regular supply of food and to provide for their personal safety in some cases than in others, can only be balanced by a difference in the population which have to exist in a given area—we shall be in a condition to proceed to the consideration of varieties, to which the preceding remarks have a direct and very important application. http://darwin-online.org.uk/content/frameset?pageseq=1&itemID=F1700&viewtype=text
38. Malthus on man should be studied; and all such cases as those of the mice in La Plata, of the cattle and horses when first turned out in S. America, of the robins by our calculation, &c., should be well considered: reflect on the enormous multiplying power inherent and annually in action in all animals; reflect on the countless seeds scattered by a hundred ingenious contrivances, year after year, over the whole face of the land; and yet we have every reason to suppose that the average percentage of every one of the inhabitants of a country will ordinarily remain constant. Finally, let it be borne in mind that this average number of individuals (the external conditions remaining the same) in each country is kept up by recurrent struggles against other species or against external nature (as on the borders of the arctic regions2, where the cold checks life). http://darwin-online.org.uk/content/frameset?pageseq=1&itemID=F1556&viewtype=text
39. Hence this fact which seems at first paradoxical, & is so if we look chiefly to climatal or soil conditions as of predominating influence, ceases to be paradoxical when we look at all organic beings as periodically struggling for existence with their utmost energy against their enemies. All these cases show only that the struggle for existence is periodical not incessant, of which fact we have plenty of other evidence: in the first very severe winter the rooks with the crossed bills would no doubt be cleared off. lustrations of the Action of Natural Selection. In order to make it clear how I believe natural selection acts, I must beg permission to give one or two imaginary illustrations./12/Let us take the case of a wolf, which preys on various animals, securing some by craft, some by strength & some by fleetness; & let us suppose that the fleetest prey, a deer for instance, had from any change whatever increased in numbers, or other prey had decreased in numbers during that season of the year, when the wolf is hardest pressed for food; I can under such circumstances see no/<12>13/reason to doubt that the swiftest & slimmest wolves would in the long run be preserved & selected; always provided that they retained strength to master their prey at this period or some other period of the year when compelled to prey on other animals./13 v/I can see no more reason to doubt this, than

that the Breeder can greatly improve the fleetness of his greyhounds by long-continued & careful selection. /13/The same process would tend to modify the deer in order to escape the wolf slowly rendered fleeter; though it might happen that some other & incompatable modification might be more important to this animal, as getting food during some other season for the destruction may fall before an habitually recurrent period of dearth, which would have in any case thinned their numbers: it is even quite conceivable that such destruction might increase the minimum average, for more food might thus be preserved against the period of dearth, as for instance in dry countries, in which the herbage withers up & serves as natural hay. Many other considerations might have been added showing how complex the action & reaction of the checks to increase must be. http://darwin-online.org.uk/content/frameset?pageseq=1&itemID=F1583&viewtype=text

40. DeCandolle, in an eloquent passage, has declared that all nature is at war, one organism with another, or with external nature. Seeing the contented face of nature, this may at first well be doubted; but reflection will inevitably prove it to be true. The war, however, is not constant, but recurrent in a slight degree at short periods, and more severely at occasional more distant periods; and hence its effects are easily overlooked. It is the doctrine of Malthus applied in most cases with tenfold force. As in every climate there are seasons, for each of its inhabitants, of greater and less abundance, so all annually breed; and the moral restraint which in some small degree checks the increase of mankind is entirely lost. Even slow-breeding mankind has doubled in twenty-five years; and if he could increase his food with greater ease, he would double in less time. But for animals without artificial means, the amount of food for each species must, on an average, be constant, whereas the increase of all organisms tends to be geometrical, and in a vast majority of cases at an enormous ratio. Suppose in a certain spot there are eight pairs of birds, and that only four pairs of them annually (including double hatches) rear only four young, and that these go on rearing their young at the same rate, then at the end of seven years (a short life, excluding violent deaths, for any bird) there will be 2048 birds, instead of the original sixteen. As this increase is quite impossible, we must conclude either that birds do not rear nearly half their young, or that the average life of a bird is, from accident, not nearly seven years. Both checks probably concur. The same kind of calculation applied to all plants and animals affords results more or less striking, but in very few instances more striking than in man. Finally, let it be borne in mind that this average number of individuals (the external conditions remaining the same) in each country is kept up by recurrent struggles against other species or against external nature (as on the borders of the Arctic regions, where the cold checks life), and that ordinarily each individual of every species holds its place, either by its own struggle and capacity of acquiring nourishment in some period of its life, from the egg upwards; or by the struggle of its parents (in short-lived organisms, when the main check occurs at longer intervals) with other individuals of the same or different species. Those whose organization does not permit them to migrate when their food becomes periodically scarce, can never attain a large population. This is probably the reason why woodpeckers are scarce with us, while in the tropics they arc among the most abundant of solitary birds. Thus the house sparrow is more abundant than the redbreast, because its food is more constant and plentiful,—seeds of grasses being preserved during the winter, and our farm-yards and -stubble-fields furnishing an almost inexhaustible supply. Why, as a general rule, are aquatic, and especially sea birds, very numerous in individuals? Not because they are more prolific than others, generally the contrary; but because their food never fails, the sea-shores and river-banks daily swarming with a fresh supply of small Mollusca and Crustacea. Exactly the same laws will apply to mammals. Wild cats are prolific and have few enemies; why then are they never as abundant as rabbits? The only intelligible answer is, that their supply of food is more precarious. It appears evident, therefore, that so long as a country remains physically unchanged, the numbers of its animal population cannot materially increase. If one species does so, some others requiring the same kind of food must diminish in proportion. The numbers that die

annually must be immense; and as the individual existence of each animal depends upon itself, those that die must be the weakest—the very young, the aged, and the diseased,—while those that prolong their existence can only be the most perfect in health and vigour—those who are best able to obtain food regularly, and avoid their numerous enemies. It is, as we commenced by remarking, "a struggle for existence," in which the weakest and least perfectly organized must always succumb. http://darwin-online.org.uk/content/frameset?pageseq=1&itemID=F1700&viewtype=text

Ancestral rhythms

41. The inhabitants of the seashore must be greatly affected by the tides; animals living either about the mean high-water mark, or about the mean low-water mark, pass through a complete cycle of tidal changes in a fortnight. Consequently, their food supply will undergo marked changes week by week. The vital functions of such animals, living under these conditions for many generations, can hardly fail to run their course in regular weekly periods. Now it is a mysterious fact that in the higher and now terrestrial Vertebrata, as well as in other classes, many normal and abnormal processes have one or more whole weeks as their periods; this would be rendered intelligible if the Vertebrata are descended from an animal allied to the existing tidal Ascidians. Many instances of such periodic processes might be given, as the gestation of mammals, the duration of fevers, &c. The hatching of eggs affords also a good example, for, according to Mr. Bartlett ('Land and Water,' Jan. 7, 1871), the eggs of the pigeon are hatched in two weeks; those of the fowl in three; those of the duck in four; those of the goose in five; and those of the ostrich in seven weeks. As far as we can judge, a recurrent period, if approximately of the right duration for any process or function, would not, when once gained, be liable to change; consequently it might be thus transmitted through almost any number of generations. But if the function changed, the period would have to change, and would be apt to change almost abruptly by a whole week. This conclusion, if sound, is highly remarkable; for the period of gestation in each mammal, and the hatching of each bird's eggs, and many other vital processes, thus betray to us the primordial birthplace of these animals. http://darwin-online.org.uk/content/frameset?keywords=of%20greatly%20affected%20by%20tides%20the%20must%20seashore%20be%20inhabitants&pageseq=187&itemID=F944&viewtype=text
42. All vital functions tend to run their course in fixed and recurrent periods, and with tidal animals the periods would probably be lunar; for such animals must have been left dry or covered deep with water,—supplied with copious food or stinted,—during endless generations, at regular lunar intervals. If then the Vertebrata are descended from an animal allied to the existing tidal Ascidians, the mysterious fact, that with the higher and now terrestrial Vertebrata, not to mention other classes, many normal and abnormal vital processes run their course according to lunar periods, is rendered intelligible. A recurrent period, if approximately of the right duration, when once gained, would not, as far as we can judge, be liable to be changed; consequently it might be thus transmitted during almost any number of generations. This conclusion, if it could be proved sound, would be curious; for we should then see that the period of gestation in each mammal, and the hatching of each bird's eggs, and many other vital processes, still betrayed the primordial birthplace of these animals. http://darwin-online.org.uk/content/frameset?pageseq=1&itemID=F937.1&viewtype=text
43. At a still earlier period the progenitors of man must have been aquatic in their habits; for morphology plainly tells us that our lungs consist of a modified swim-bladder, which once served as a float. The clefts on the neck in the embryo of man show where the branchiæ once existed. In the lunar or weekly recurrent periods of some of our functions we apparently still retain traces of our primordial birthplace, a shore washed by the tides. At about this same early period the true kidneys were replaced by the corpora wolffiana. The heart existed as a simple pulsating vessel; and the chorda dorsalis took the place of a vertebral column. These

early ancestors of man, thus seen in the dim recesses of time, must have been as simply, or even still more simply organised than the lancelet or amphioxus. http://darwin-online.org.uk/content/frameset?pageseq=1&itemID=F944&viewtype=text

5. Temporal isolation as a mechanism for speciation

1. Even in the case of slow-breeding animals, which unite for each birth, we must not overrate the effects of intercrosses in retarding natural selection; for I can bring a considerable catalogue of facts, showing that within the same area, varieties of the same animal can long remain distinct, from haunting different stations, from breeding at slightly different seasons, or from varieties of the same kind preferring to pair together. http://darwin-online.org.uk/content/frameset?keywords=which%20of%20in%20for%20birth%20slow%20each%20the%20case%20unite%20even%20breeding%20animals&pageseq=118&itemID=F373&viewtype=text
2. In some few cases varieties tend to keep distinct, by breeding at different periods, by great differences in size, or by sexual preference. http://darwin-online.org.uk/content/frameset?pageseq=1&itemID=F877.2&viewtype=text
3. In one specimen, the spermatozoa in the hermaphrodite and in the male were mature at the same time; in another this was not the case; and as the males, apparently, become attached at all periods of the year, this want of coincidence in maturity must often occur. Can the males retain their spermatozoa, till told by some instinct, that the ova in the sack of the often fecundated hermaphrodite are ready for impregnation; or are the spermatozoa sometimes wasted, as must annually happen with such incalculable quantities of the pollen of many diœcious plants? http://darwin-online.org.uk/content/frameset?pageseq=1&itemID=F339.1&viewtype=text
4. Nor should we forget the facts, already given, of varieties of the most freely crossing animals, sometimes keeping apart, or breeding at different seasons &c, which would greatly lessen or prevent the formation/60/of intermediate links by crossing, though it would not often lessen the function of such links in relation to the inter-mediate state of the conditions of life. http://darwin-online.org.uk/content/frameset?pageseq=1&itemID=F1583&viewtype=text
5. Intercrossing will prevent or retard the process of natural selection; but here we are involved in much doubt. Those animals, which move much about & unite for each birth will thus be kept truest to their parental type; or if undergoing change will be modified in an insensible manner, without any recognizable variety being formed at any one period. It may, however, be otherwise in those cases, in which varieties of the most freely crossing animals,/70/from their very first commencement, haunt some distinct station or breed at different periods &c. Those organisms which rarely cross, & which are capable of increasing at a quick rate, may be formed on some one spot, & thence spread with little retardation from intercrossing. http://darwin-online.org.uk/content/frameset?pageseq=1&itemID=F1583&viewtype=text
6. I have remarked that in animals of which two individuals unite at each act of reproduction some degree of separation must be if not actually necessary, yet most advantageous. This may arise from a selected individual with its descendants, as soon as formed even into an extremely slightly different variety, tending to haunt a somewhat different station, breeding at a somewhat different season, & from like varieties preferring to pair with each other. http://darwin-online.org.uk/content/frameset?pageseq=1&itemID=F1583&viewtype=text
7. Birds of the same species inhabiting the same country, some of which migrate and some do not & which can be distinguished by very slight differences. In all such cases there would be some tendency for varieties having such different habits to keep distinct. http://darwin-online.org.uk/content/frameset?pageseq=1&itemID=F1583&viewtype=text
8. With plants a difference in the period of flowering serves to keep varieties distinct, as with the various kinds of maize and wheat: thus Colonel Le Couteur remarks, "the Talavera wheat, from flowering much earlier than any other kind, is sure to continue pure.

http://darwin-online.org.uk/content/frameset?keywords=to%20of%20in%20a%20the%20difference%20plants%20serves%20distinct%20flowering%20period%20keep%20with%20varieties&pageseq=116&itemID=F878.2&viewtype=text

9. Cross-fertilisation is sometimes ensured by the sexes being separated, and in a large number of cases by the pollen and stigma of the same flower being matured at different times. http://darwin-online.org.uk/content/frameset?pageseq=1&itemID=F1249&viewtype=text
10. The flowers were incessantly visited by bees, so that the stigmas must have received on the most favourable days, and at the most favourable hours, successive applications of pollen: all who have crossed plants know that this highly favours fertilization. . http://darwin-online.org.uk/content/frameset?pageseq=1&itemID=F1731&viewtype=text
11. Whether or not the dimorphic condition of the Primulæ has any bearing on other points in natural history, it is valuable as showing how nature strives, if I may so express myself, to favour the sexual union of distinct individuals of the same species. The resources of nature are illimitable; and we know not why the species of Primula should have acquired this novel and curious aid for checking continued self-fertilization through the division of the individuals into two bodies of hermaphrodites with different sexual powers, instead of by the more common method of the separation of the sexes, or by the maturity of the male and female elements at different periods, or by other such contrivances. . http://darwin-online.org.uk/content/frameset?pageseq=1&itemID=F1717&viewtype=text

6. Inheritance and endogenous nature of periodical phenomena

1. I will not attempt any definition of instinct. It would be easy to show that several distinct mental actions are commonly embraced by this term; but every one understands what is meant, when it is said that instinct impels the cuckoo to migrate and to lay her eggs in other birds nests. An action, which we ourselves should require experience to enable us to perform, when performed by an animal, more especially by a very young one, without any experience, and when performed by many individuals in the same way, without their knowing for what purpose it is performed, is usually said to be instinctive. [...] Changes of instinct may sometimes be facilitated by the same species having different instincts at different periods of life, or at different seasons of the year, or when placed under different circumstances, &c.; in which case either one or the other instinct might be preserved by natural selection. And such instances of diversity of instinct in the same species can be shown to occur in nature. [...] I can only assert, that instincts certainly do vary—for instance, the migratory instinct, both in extent and direction, and in its total loss. So it is with the nests of birds, which vary partly in dependence on the situations chosen, and on the nature and temperature of the country inhabited, but often from causes wholly unknown to us. [...] A number of curious and authentic instances could be given of the inheritance of all shades of disposition and tastes, and likewise of the oddest tricks, associated with certain frames of mind or periods of time. [...] It is now commonly admitted that the more immediate and final cause of the cuckoos instinct is, that she lays her eggs, not daily, but at intervals of two or three days; so that, if she were to make her own nest and sit on her own eggs, those first laid would have to be left for some time unincubated, or there would be eggs and young birds of different ages in the same nest. If this were the case, the process of laying and hatching might be inconveniently long, more especially as she has to migrate at a very early period; and the first hatched young would probably have to be fed by the male alone. But the American cuckoo is in this predicament; for she makes her own nest and has eggs and young successively hatched, all at the same time.
https://darwin-online.org.uk/content/frameset?itemID=F376&keywords=authentic%20of%20could%20be%20the%20instances%20given%20all%20shades%20inheritance%20dispositions&viewtype=text&pageseq=231

2. The mental powers of different animals in wild and tame state require a separate section. Be it remembered I have nothing to do with origin of memory, attention, and the different faculties of the mind, but merely with their differences in each of the great divisions of nature. Disposition, courage, pertinacity, suspicion, restlessness, ill-temper, sagacity and the reverse unquestionably vary in animals and are inherited. Habits purely corporeal, breeding season &c., time of going to rest &c., vary and are hereditary, like the analogous habits of plants which vary and are inherited. Habits of body, as manner of movement. Habits, as pointing and setting on certain occasions. Taste for hunting certain objects and manner of doing so. These are shown clearly by crossing and their analogy with true instinct thus shown. Do not know objects for which they do it. Lord Brougham's definition. Origin partly habit, but the amount necessarily unknown, partly selection. Young pointers pointing stones and sheep—tumbling pigeons—sheep going back to place where born. https://darwin-online.org.uk/content/frameset?itemID=F1555&keywords=of%20going%20time%20rest%20to&viewtype=text&pageseq=47
3. The "transandantes" sheep in Spain, which for some centuries have been yearly taken a journey of several hundred miles from one province to another, know when the time comes, and show the greatest restlessness (like migratory birds in confinement), and are prevented with difficulty from starting by themselves, which they sometimes do, and find their own way. There is a case on good evidence of a sheep which, when she lambed, would return across a mountainous country to her own birth-place, although at other times of year not of a rambling disposition. Her lambs inherited this same disposition, and would go to produce their young on the farm whence their parent came; and so troublesome was this habit that the whole family was destroyed. These facts must lead to the conviction, justly wonderful as it is, that almost infinitely numerous shades of disposition, of tastes, of peculiar movements, and even of individual actions, can be modified or acquired by one individual and transmitted to its offspring..
http://darwin-online.org.uk/content/frameset?keywords=the%20spain%20in%20transandantes%20sheep&pageseq=160&itemID=F1556&viewtype=text
4. Bechstein says that from many years' experience he is certain that in the nightingale a tendency to sing in the middle of the night or in the day runs in families is strictly inherited. http://darwin-online.org.uk/content/frameset?keywords=that%20many%20from%20bechstein%20years%20says&pageseq=533&itemID=F1583&viewtype=text
5. Inheritance at corresponding Seasons of the Year.—With animals in a state of nature, innumerable instances occur of characters appearing periodically at different seasons. We see this in the horns of the stag, and in the fur of arctic animals which becomes thick and white during the winter. Many birds acquire bright colours and other decorations during the breeding-season alone. Pallas states that in Siberia domestic cattle and horses become lighter-coloured during the winter; and I have myself observed, and heard of similar strongly marked changes of colour, that is, from brownish cream-colour or reddish-brown to a perfect white, in several ponies in England. Although I do not know that this tendency to change the colour of the coat during different seasons is transmitted, yet it probably is so, as all shades of colour are strongly inherited by the horse. Nor is this form of inheritance, as limited by the seasons, more remarkable than its limitation by age or sex. http://darwin-online.org.uk/content/frameset?keywords=the%20at%20of%20corresponding%20inheritance%20seasons%20year&pageseq=295&itemID=F937.1&viewtype=text
6. But the most curious instance known to me of one instinct conquering another, is the migratory instinct conquering the maternal instinct. The former is wonderfully strong; a confined bird will at the proper season beat her breast against the wires of her cage, until it is bare and bloody. It causes young salmon to leap out of the fresh water, where they could still continue to live, and thus unintentionally to commit suicide. Everyone knows how strong the maternal instinct is, leading even timid birds to face great danger, though with hesitation and in opposition to the instinct of self-preservation. Nevertheless the migratory instinct is so powerful that late in the autumn swallows and house-martins frequently desert their tender young, leaving them to perish miserably in their nests.

http://darwin-online.org.uk/content/frameset?keywords=most%20one%20curious%20to%20of%20the%20instinct%20instance%20me%20but%20known&pageseq=96&itemID=F937.1&viewtype=text

7. Man is subject like other mammals, birds, and even insects, to that mysterious law, which causes certain normal processes, such as gestation, as well as the maturation and duration of various diseases, to follow lunar periods. http://darwin-online.org.uk/content/frameset?keywords=birds%20is%20subject%20mammals%20man%20other%20like&pageseq=25&itemID=F937.1&viewtype=text
8. In the migration of animals, the instinct which impels them to proceed in a certain direction ought, I think, to be distinguished from the unknown means by which they can tell one direction from another & by which, after starting, they are enabled to keep their course in a dark night over the open sea; & likewise from the means, whether some instinctive association with changing temperature or with want of food &c, which leads them to start at the proper period. In this, & other cases, the several parts of the problem have often been confounded together under the word instinct. With respect to the period of starting; it cannot of course be memory, as young Cuckoos start for the first time two months after their parents have departed: yet it deserves notice that animals somehow acquire a surprisingly accurate idea of time: A. d'Orbigny shows that a lame Caracara Hawk in S. America, knew the period of three weeks & used at this interval to visit monasteries where food was distributed to the poor. Difficult though it may be to conceive how animals either intelligently or instinctively come to know a given period; yet we shall immediately see that in some cases, our domestic animals have acquired an annually recurring impulse to travel, extremely like, if not identical with, a true migratory instinct; & which can hardly be due to mere memory. It is a true instinct which leads the pinioned Brent goose to try to escape northward; but how the bird distinguishes north & south we know not. Nor do we know how a bird which starts in the night as many do, to traverse the ocean, keeps its course, as if provided with a compass. But we should be very cautious in attributing to migratory animals any capacity in this respect, which we do not ourselves possess; though certainly in them carried to a wonderful perfection. http://darwin-online.org.uk/content/frameset?keywords=migration%20which%20to%20direction%20of%20proceed%20in%20ought%20a%20impels%20the%20instinct%20them%20certain%20animals&pageseq=10&itemID=F1434&viewtype=text
9. Pfeffer denies such inheritance; he attributes the periodicity when prolonged for a day or two in darkness, to "Nachwirkung," or the after-effects of light and darkness. But we are unable to follow his train of reasoning. There does not seem to be any more reason for attributing such movements to this cause than, for instance, the inherited habit of winter and summer wheat to grow best at different seasons; for this habit is lost after a few years, like the movements of leaves in darkness after a few days. No doubt some effect must be produced on the seeds by the long-continued cultivation of the parent-plants under different climates, but no one probably would call this the "Nachwirkung" of the climates. http://darwin-online.org.uk/content/frameset?keywords=pfeffer%20denies%20inheritance%20such&pageseq=1&itemID=CUL-DAR209.7.153&viewtype=text
10. Animals can certainly by some means judge the intervals of time between recurrent events. http://darwin-online.org.uk/content/frameset?keywords=certainly%20some%20can%20by%20judge%20means%20animals&pageseq=58&itemID=F937.1&viewtype=text
11. Thirdly, in considering many instincts it is useful to endeavour to separate the faculty by which they perform it, and the mental power which urges to the performance, which is more properly called an instinct. We have an instinct to eat, we have jaws &c. to give us the faculty to do so. These faculties are often unknown to us: bats, with their eyes destroyed, can avoid strings suspended across a room, we know not at present by what faculty they do this. Thus also, with migratory birds, it is a wonderful instinct which urges them at certain times of the year to direct their course in certain directions, but it is a faculty by which they know the time and find their way. With respect to time, man without seeing the sun can judge to a certain extent of

the hour, as must those cattle which come down from the inland mountains to feed on sea-weed left bare at the changing hour of low-water. A hawk (D'Orbigny) seems certainly to have acquired a knowledge of a period of every 21 days. In the cases already given of the sheep which travelled to their birth-place to cast their lambs, and the sheep in Spain which know their time of march, we may conjecture that the tendency to move is associated, we may then call it instinctively, with some corporeal sensations. With respect to direction we can easily conceive how a tendency to travel in a certain course may possibly have been acquired, although we must remain ignorant how birds are able to preserve any direction whatever in a dark night over the wide ocean. I may observe that the power of some savage races of mankind to find their way, although perhaps wholly different from the faculty of birds, is nearly as unintelligible to us. Bellinghausen, a skilful navigator, describes with the utmost wonder the manner in which some Esquimaux guided him to a certain point, by a course never straight, through newly formed hummocks of ice, on a thick foggy day, when he with a compass found it direction: so it is with Australian savages in thick forests. In North and South America many birds slowly travel northward and southward, urged on by the food they find, as the seasons change; let them continue to do this, till, as in the case of the sheep in Spain, it has become an urgent instinctive desire, and they will gradually accelerate their journey. They would cross narrow rivers, and if these were converted by subsidence into narrow estuaries, and gradually during centuries to arms of the sea, still we may suppose their restless desire of travelling onwards would impel them to cross such an arm, even if it had become of great width beyond their span of vision. How they are able to preserve a course in any direction, I have said, is a faculty unknown to us. To give another illustration of the means by which I conceive it possible that the direction of migrations have been determined. Elk and reindeer in N. America annually cross, as if they could marvellously smell or see at the distance of a hundred miles, a wide tract of absolute desert, to arrive at certain islands where there is a scanty supply of food; the changes of temperature, which geology proclaims, render it probable that this desert tract formerly supported some vegetation, and thus these quadrupeds might have been annually led on, till they reached the more fertile spots, and so acquired, like the sheep of Spain, their migratory powers. http://darwin-online.org.uk/content/frameset?keywords=is%20useful%20which%20to%20instincts%20in%20separate%20they%20endeavour%20by%20considering%20it%20the%20faculty%20many%20thirdly&pageseq=159&itemID=F1556&viewtype=text

12. The chief characteristics of true instincts appear to be their invariability and non-improvement during the mature age of the individual animal: the absence of knowledge of the end, for which the action is performed, being associated, however, sometimes with a degree of reason; being subject to mistakes and being associated with certain states of the body or times of the year or day…This last fact offers an instance of a domestic instinct being associated with a state of body; as do the "transandantes" sheep with a time of year. http://darwin-online.org.uk/content/frameset?keywords=is%20useful%20which%20to%20instincts%20in%20separate%20they%20endeavour%20by%20considering%20it%20the%20faculty%20many%20thirdly&pageseq=159&itemID=F1556&viewtype=text
13. The term instinct is often used in <a> sense which implies no more than that the animal does the action in question. Faculties and instincts may I think be imperfectly separated. The mole has the faculty of scratching burrows, and the instinct to apply it. The bird of passage has the faculty of finding its way and the instinct to put it in action at certain periods. It can hardly be said to have the faculty of knowing the time, for it can possess no means, without indeed it be some consciousness of passing sensations. Think over all habitual actions and see whether faculties and instincts can be separated. We have faculty of waking in the night, if an instinct prompted us to do something at certain hour of night or day. Savages finding their way. Wrangel's account—probably a faculty inexplicable by the possessor. There are besides faculties "means," as conversion of larvae into neuters and queens. http://darwin-online.org.uk/content/frameset?keywords=is%20useful%20which%20to%20insti

ncts%20in%20separate%20they%20endeavour%20by%20considering%20it%20the%20faculty%20many%20thirdly&pageseq=159&itemID=F1556&viewtype=text

7. A pioneer for chronobiological experiments?

1. Worms are nocturnal in their habits, and at night may be seen crawling about in large numbers, but usually with their tails still inserted in their burrows . http://darwin-online.org.uk/content/frameset?keywords=at%20may%20and%20night%20in%20are%20habits%20crawling%20be%20seen%20worms%20their%20nocturnal&pageseq=25&itemID=F1361&viewtype=text
2. During the day they remain in their burrows, except at the pairing season, when those which inhabit adjoining burrows expose the greater part of their bodies for an hour or two in the early morning. http://darwin-online.org.uk/content/frameset?keywords=at%20may%20and%20night%20in%20are%20habits%20crawling%20be%20seen%20worms%20their%20nocturnal&pageseq=25&itemID=F1361&viewtype=text
3. The period during which worms near Calcutta display such extraordinary activity lasts for only a little over two months, namely, during the cool season after the rains. At this time they are generally found within about 10 inches beneath the surface. During the hot season they burrow to a greater depth, and are then found coiled up and apparently hybernating. Mr. Scott has never seen them at a greater depth than 2½ feet, but has heard of their having been found at 4 feet. Within the forests, fresh castings may be found even during the hot season. The worms in the Botanic garden, during the cool and dry season, draw many leaves and little sticks into the mouths of their burrows, like our English worms; but they rarely act in this manner during the rainy season . http://darwin-online.org.uk/content/frameset?keywords=at%20may%20and%20night%20in%20are%20habits%20crawling%20be%20seen%20worms%20their%20nocturnal&pageseq=25&itemID=F1361&viewtype=text
4. Pieces of raw and roasted meat were fixed several times by long pins to the surface of the soil in my pots, and night after night the worms could be seen tugging at them, with the edges of the pieces engulfed in their mouths, so that much was consumed . http://darwin-online.org.uk/content/frameset?keywords=at%20may%20and%20night%20in%20are%20habits%20crawling%20be%20seen%20worms%20their%20nocturnal&pageseq=25&itemID=F1361&viewtype=text
5. Why the process regularly takes place in the two anterior glands, and only rarely in the four posterior glands is quite unknown. Morren says that these glands disappear during the winter; and I have seen some instances of this fact, and others in which either the anterior or posterior glands were at this season so shrunk and empty, that they could be distinguished only with much difficulty. http://darwin-online.org.uk/content/frameset?keywords=at%20may%20and%20night%20in%20are%20habits%20crawling%20be%20seen%20worms%20their%20nocturnal&pageseq=25&itemID=F1361&viewtype=text
6. This difficulty led my son Francis and myself to observe worms in confinement during several nights by the aid of a dim light, while they dragged the leaves of the above named pines into their burrows. http://darwin-online.org.uk/content/frameset?keywords=at%20may%20and%20night%20in%20are%20habits%20crawling%20be%20seen%20worms%20their%20nocturnal&pageseq=25&itemID=F1361&viewtype=text
7. From the foregoing facts it is evident that light affects worms by its intensity and by its duration. It is only the anterior extremity of the body, where the cerebral ganglia lie, which is affected by light, as Hoffmeister asserts, and as I observed on many occasions. If this part is shaded, other parts of the body may be fully illuminated, and no effect will be produced. As

these animals have no eyes, we must suppose that the light passes through their skins, and in some manner excites their cerebral ganglia. It appeared at first probable that the different manner in which they were affected on different occasions might be explained, either by the degree of extension of their skin and its consequent transparency, or by some particular incidence of the light; but I could discover no such relation. http://darwin-online.org.uk/content/frameset?keywords=at%20may%20and%20night%20in%20are%20habits%20crawling%20be%20seen%20worms%20their%20nocturnal&pageseq=25&itemID=F1361&viewtype=text

8. When the light from a candle was concentrated by means of a large lens on the anterior extremity, they generally withdrew instantly; but this concentrated light failed to act perhaps once out of half a dozen trials. The light was on one occasion concentrated on a worm lying beneath water in a saucer, and it instantly withdrew into its burrow. In all cases the duration of the light, unless extremely feeble, made a great difference in the result; for worms left exposed before a paraffin lamp or a candle invariably retreated into their burrows within from five to fifteen minutes; and if in the evening the pots were illuminated before the worms had come out of their burrows, they failed to appear. http://darwin-online.org.uk/content/frameset?keywords=at%20may%20and%20night%20in%20are%20habits%20crawling%20be%20seen%20worms%20their%20nocturnal&pageseq=25&itemID=F1361&viewtype=text
9. But the different effect which a light produced on different occasions, and especially the fact that a worm when in any way employed and in the intervals of such employment, whatever set of muscles and ganglia may then have been brought into play, is often regardless of light, are opposed to the view of the sudden withdrawal being a simple reflex action. With the higher animals, when close attention to some object leads to the disregard of the impressions which other objects must be producing on them, we attribute this to their attention being then absorbed; and attention implies the presence of a mind. Every sportsman knows that he can approach animals whilst they are grazing, fighting or courting, much more easily than at other times. The state, also, of the nervous system of the higher animals differs much at different times, for instance, a horse is much more readily startled at one time than at another. The comparison here implied between the actions of one of the higher animals and of one so low in the scale as an earth-worm, may appear far-fetched; for we thus attribute to the worm attention and some mental power, nevertheless I can see no reason to doubt the justice of the comparison.
http://darwin-online.org.uk/content/frameset?keywords=at%20may%20and%20night%20in%20are%20habits%20crawling%20be%20seen%20worms%20their%20nocturnal&pageseq=25&itemID=F1361&viewtype=text
10. They often showed their sensitiveness when the pot in which they lived, or the table on which the pot stood, was accidentally and lightly struck; but they appeared less sensitive to such jars than to the vibrations of the piano; and their sensitiveness to jars varied much at different times.https://darwin-online.org.uk/content/frameset?itemID=F1357&keywords=in%20the%20showed%20when%20sensitiveness%20their%20often%20pot%20they%20lived%20which&viewtype=text&pageseq=42
11. Although worms cannot be said to possess the power of vision, their sensitiveness to light enables them to distinguish between day and night; and they thus escape extreme danger from the many diurnal animals which prey on them. Their withdrawal into their burrows during the day appears, however, to have become an habitual action; for worms kept in pots covered by glass-plates, over which sheets of black paper were spread, and placed before a north-east window, remained during the day-time in their burrows and came out every night; and they continued thus to act for a week. No doubt a little light may have entered between the sheets of glass and the blackened paper; but we know from the trials with coloured glass, that worms are indifferent to a small amount of light. http://darwin-online.org.uk/content/frameset?keywords=at%20may%20and%20night%20in%2

0are%20habits%20crawling%20be%20seen%20worms%20their%20nocturnal&pageseq=25&
itemID=F1361&viewtype=text
