# Supplementary figures and images for: Darwin and the biological rhythms
Source: PNAS Nexus. 2024 Aug 27;3(8):pgae318. doi: 10.1093/pnasnexus/pgae318 (PMC11348560; doi:10.1093/pnasnexus/pgae318)

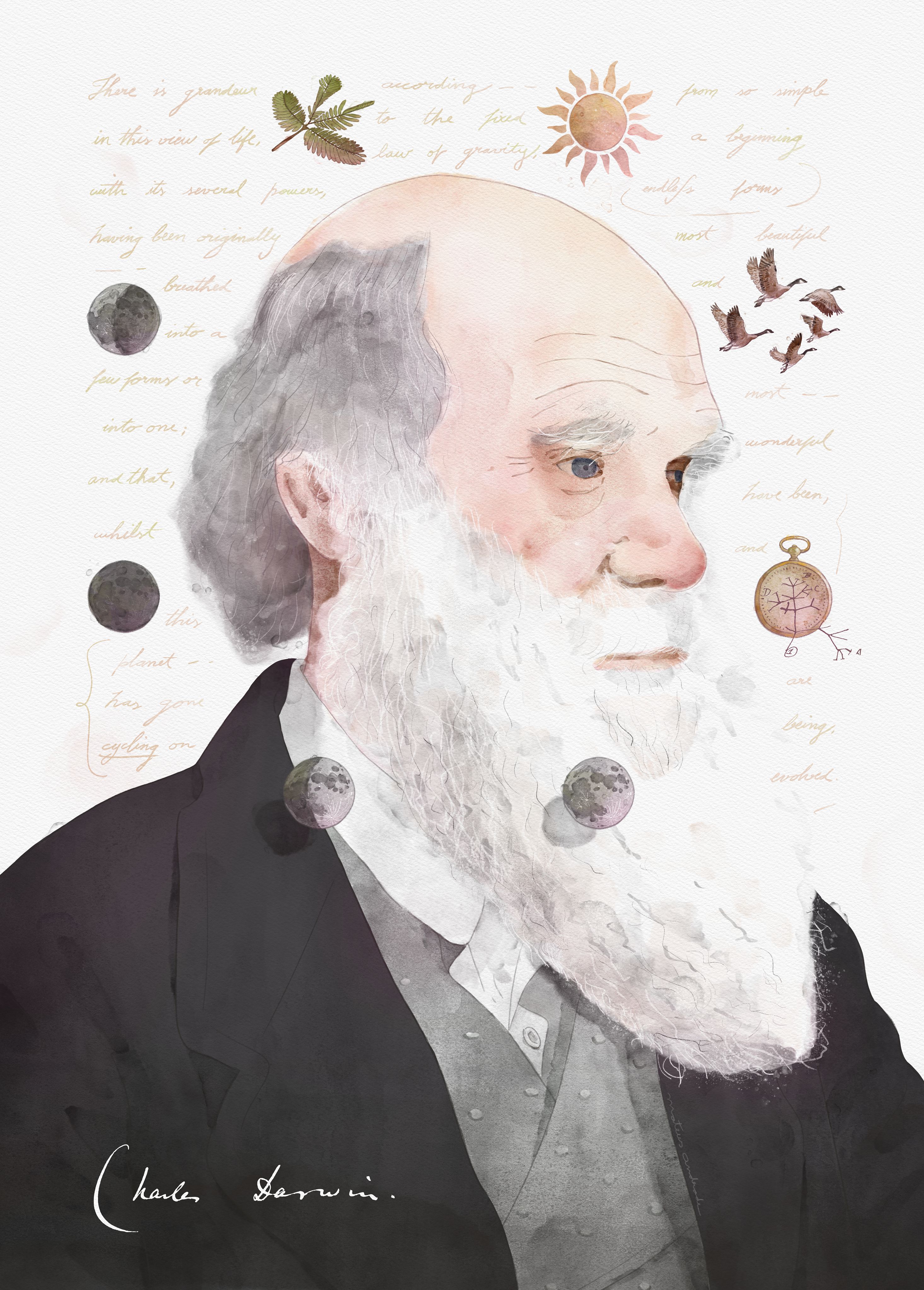

Supplement: pgae318_Supplementary_Data [file pgae318_supplementary_data.zip › A4 350dpi.jpg]
